# Representation of edges, head direction, and swimming kinematics in the brain of freely-navigating fish

**DOI:** 10.1038/s41598-020-71217-1

**Published:** 2020-09-08

**Authors:** Ehud Vinepinsky, Lear Cohen, Shay Perchik, Ohad Ben-Shahar, Opher Donchin, Ronen Segev

**Affiliations:** 1 Department of Life Sciences, Ben Gurion University of the Negev, 84105 Beer Sheva, Israel; 2 Zlotowski Center for Neuroscience, Ben Gurion University of the Negev, 84105 Beer Sheva, Israel; 3 Department of Biomedical Engineering, Ben Gurion University of the Negev, 84105 Beer Sheva, Israel; 4 Department of Cognitive and Brain Sciences, Ben-Gurion University of the Negev, 84105 Beer Sheva, Israel; 5 Department of Computer Sciences, Ben Gurion University of the Negev, 84105 Beer Sheva, Israel

**Keywords:** Neuroscience, Spatial memory

## Abstract

Like most animals, the survival of fish depends on navigation in space. This capacity has been documented in behavioral studies that have revealed navigation strategies. However, little is known about how freely swimming fish represent space and locomotion in the brain to enable successful navigation. Using a wireless neural recording system, we measured the activity of single neurons in the goldfish lateral pallium, a brain region known to be involved in spatial memory and navigation, while the fish swam freely in a two-dimensional water tank. We found that cells in the lateral pallium of the goldfish encode the edges of the environment, the fish head direction, the fish swimming speed, and the fish swimming velocity-vector. This study sheds light on how information related to navigation is represented in the brain of fish and addresses the fundamental question of the neural basis of navigation in this group of vertebrates.

## Introduction

Navigation is a fundamental behavioral capacity facilitating survival in many animal species^1–4^. It involves the continuous estimation and representation of the animal's position and direction in the environment, which are implemented in the planning and execution of movements and trajectories towards target locations^5,6^. Navigation has been extensively investigated in numerous taxa across the animal kingdom, but attempts to probe its neural substrate have mainly been focused on mammals^7^ and insects^8^. In mammals, neurons in the hippocampal formation encode information about the position and orientation of the animal in space^5–7,9,10^. These cells include place cells^11^, grid cells^12^, head direction cells^13,14^, and other cell types^15,16^. In insects, a ring-shaped neural network in the central complex of the fruit fly was shown to represent its heading direction^8^.

Teleost fish, which form the largest vertebrate class, have shown to have many high cognitive abilities with navigation among them^17–23^. To better understand space representation in non-mammalian vertebrates, we explored the neural substrate of navigation in the goldfish (*Carassius auratus*) as a representative of the teleost class. These fish are known to be able to navigate by exploiting either an allocentric or an egocentric frame of reference^24^. This may imply that the goldfish has the ability to build an internal representation of space in the form of a cognitive map^25^. This would include cognitive map-like navigation strategies to find a goal when starting from an unfamiliar initial position or taking shorter alternative routes (shortcuts) when possible^25–28^. Furthermore, goldfish integrate many environmental cues when navigating^29–31^; therefore, a change of a single cue does not impair their navigation ability in known environment^27,29^.

Previous work by Canfield and Mizumori describe a method for extracellular recording system in tethered goldfish. Their paper provides preliminary evidence for speed and spatial encoding in the goldfish lateral pallium^32^. In addition, lesion studies in goldfish have shown that the fish pallium, which is the dorsal part of the telencephalon, is crucial for spatial navigation. A lesion in the lateral areas of the pallium leads to impairment in allocentric spatial memory and learning, but not when the lesion affects other parts of the telencephalon^27^. These findings are similar to results from lesions studies of the hippocampus in mammals and further strengthen the notion that the lateral pallium in goldfish is a possible homologue of the mammalian hippocampus^25,33,34^ (but see also^35^). The goal of this study is to characterize the representation of spatial information in the teleost pallium.

## Results

**Figure 1:**
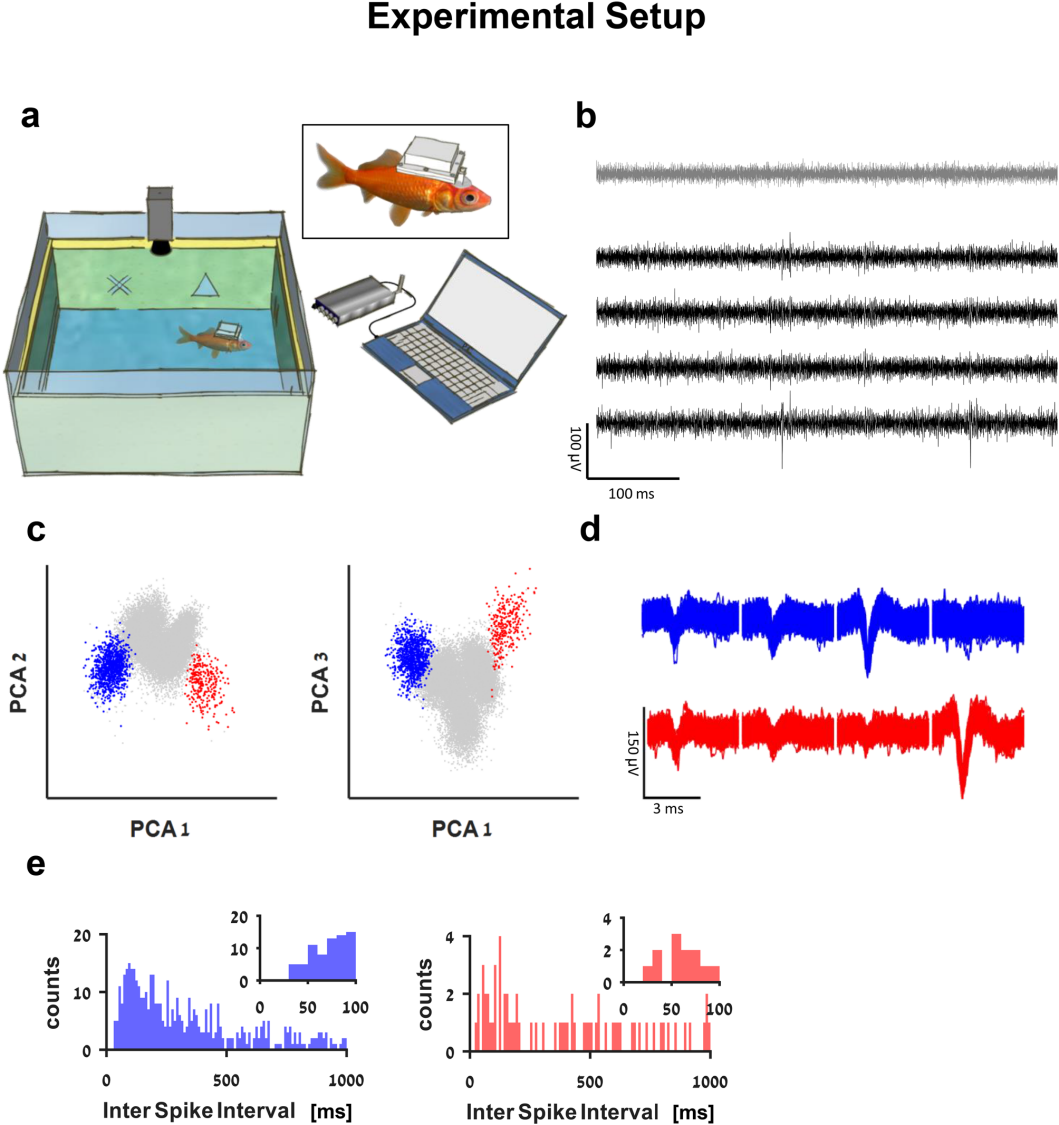
The experimental setup. (**a**) Schematic overview of the experimental setup: a fish swims freely in a water tank with electrodes, and a wireless data logger mounted on its head. The fish's movements are recorded by a video camera positioned above the tank. The tank's walls were coated with yellow foam sheets depicting different visual landmarks. (**b**) Example of a raw recording from a tetrode (black traces) and a reference electrode (gray) in the fish's lateral pallium. Neural activity can be seen in the tetrode alone. (**c**) Projection on the first three principal components of the data from the tetrode of all spike candidates that crossed the threshold. (**d**) Waveforms of two neurons after spike sorting. Other clusters were not distinguishable from other multiunit activity and neural noise. The blue cluster forms a velocity-vector cell, and the red cluster forms a head direction cell (additional examples can be found in Supplementary Figure S1a–h). (**e**) Inter spike interval histogram of the detected clusters. Insets show there were no violations of the refractory period.

In order to characterize the encoding of space and locomotion in the goldfish lateral pallium, we measured single cell activity in the lateral pallium of freely behaving goldfish while they explored a water tank. We first trained the fish (13–15 cm in body length) to swim in a shallow tank measuring 0.6 m × 0.6 m × 0.2 m (Fig. 1a). We trained the fish in seven sessions; each session was performed on a different day and lasted 20 min. After the fish became familiar with the water tank and learned to explore its entire environment naturally, we installed a recording system on their heads^36,37^. We then let the goldfish swim freely, while a camera positioned over the water tank recorded the fish's locations and head orientations. A data logger placed in a waterproof case mounted on the fish's skull recorded the activity of single cells using tetrodes (Fig. 1, see “Methods”).

Using this methodology, we identified four encoding schemes that may constitute the primitives of the goldfish navigation system: edge encoding cells, head direction encoding cells, speed correlated cells, and conjunction of head direction and speed encoding cells. Edge encoding was found in cells that were active when the fish was in close proximity to the walls of the water tank. Head direction encoding was found in cells that were more active when the fish's head was at a specific orientation. Speed correlation was found in cells with activity that was correlated with the fish swimming speed, regardless of the swimming direction. Velocity–vector correlation was found in cells that were more active when the fish swam in a particular direction and speed, thus conjugating head direction and speed characteristics.

**Figure 2:**
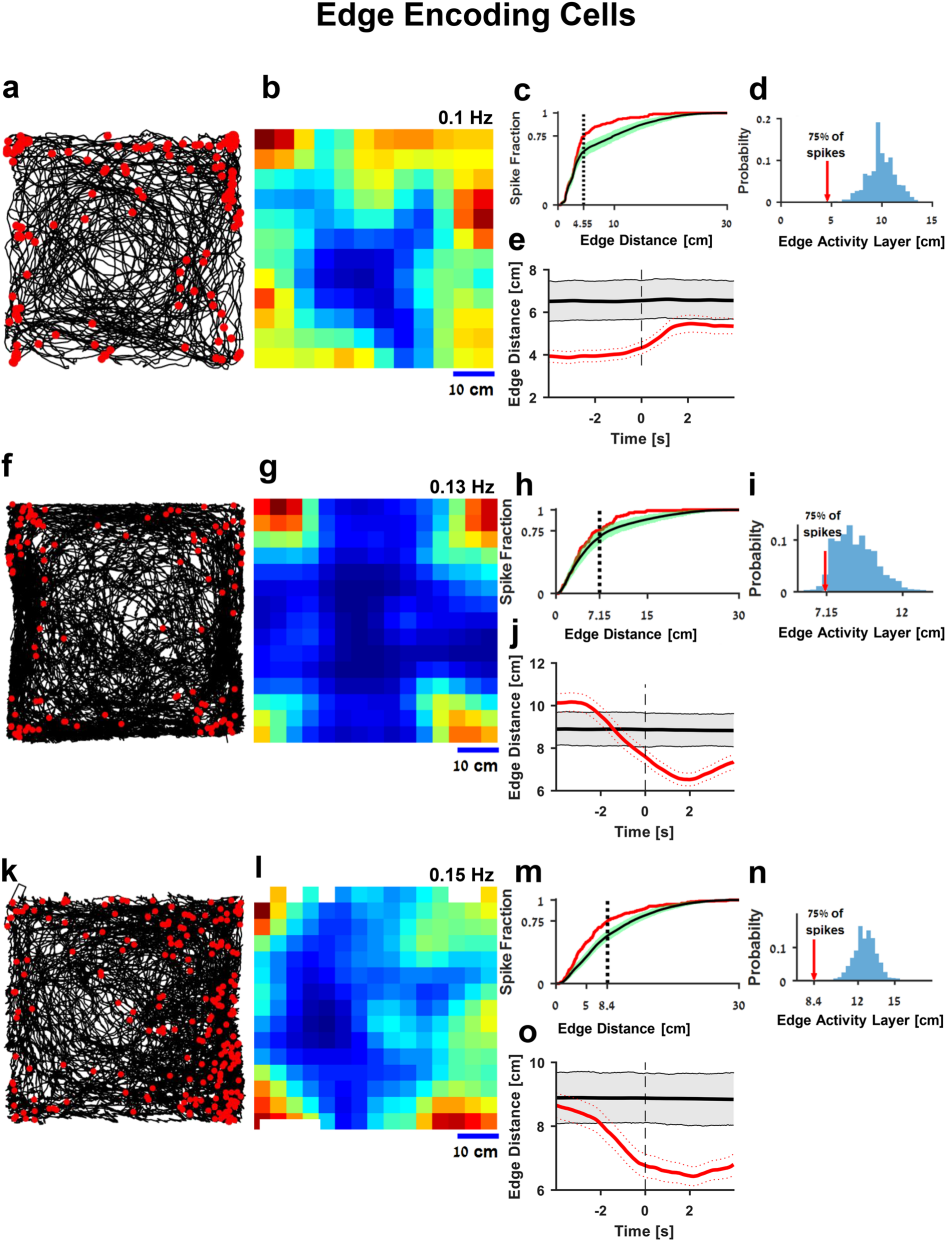
Edge encoding cells in the goldfish lateral pallium. (**a**) An example of an edge encoding cell. The fish trajectory (black curve) is presented together with the location of the fish when each spike of a single cell occurred (red dots). The apparent pattern shows that this neuron was mainly active when the fish was near the edges of the tank. The waveform of the cell spiking activity is presented in Supplementary Figure S1c. (**b**) Firing rate map of the cell in (**a**). The highest spiking probabilities are concentrated at the edges. (**c**,**d**) Statistical analysis of firing near the edge of the water tank. The red arrow (**d**) represents the edge activity layer of the recorded neuron, and the histogram shows the result of the calculated edge activity layers of 5,000 shuffled spike trains. The comparison of the red arrow and the histogram thus represents the statistical significance of the result. (**c**) Spike distribution vs. distance from the edges of the cell (red curve) and shuffled data (mean and 95% confidence interval, black and green curves) (**d**) Edge activity layer of the recorded neuron (red arrow) compared to the shuffled data (blue histogram). The edge activity layer is defined as the distance from the edge in which 75% of spikes occurred. (**e**) Spike triggered average of the fish's edge distance (red) superimposed on the 95% of shuffled data spike triggered average. (**f**–**o**) Two more examples of edge cells (additional examples are presented in Supplementary Figure S2).

Examination of the neural activity of an edge encoding cell (red dots, Fig. 2a) over the fish's trajectory (black curve) revealed a clear pattern suggesting that this neuron was mainly active when the fish swam near the edges of the water tank. This was also documented by the heat map of that neuron (Fig. 2b), color-coded from dark blue (zero firing rate) to dark red (maximal firing rate, indicated at the top right side of the pane; occupancy corrected). To test statistically whether a neuron encoded the tank edge, we first found the minimal distance from the wall in which 75% of the spikes occurred. Then, we defined this distance as the edge activity layer (red arrow, Fig. 2d). After implementing an ISI shuffling procedure (see “Methods”), we obtained 5,000 shuffled spike trains and measured the edge activity layer of each spike train (Blue histogram, Fig. 2d, see also histogram of all spike distances in Supplementary Figure S2c). When the activity of the recorded cell was significantly closer to the environmental edges (p < 0.0125, see “Methods”), the cell was classified as an edge encoding cell. As can be seen in the graph of spike distribution vs. distance from the edges (Fig. 2c), this result did not depend exclusively on the selection of the spike fraction which defines the edge activity layer. In addition, we have computed the spike triggered average of the fish distance from the edges (see “Methods”). We found that edge encoding cells have significantly lower edge distance spike triggered average than shuffled spike train (Fig. 2e). Additional examples of edge cells are depicted in Fig. 2f–o (see Supplementary Table ST1 for all encoding schemes of these cells) and Supplementary Figure S2a.

In order to test the stability of the edge tuning across trials, and to verify that the edge encoding cells indeed encoded the presence of the fish near the arena walls rather than some specific feature of the visual scene, we conducted a control experiment in which we rotated the environment by 180°. In order to eliminate the distant visual cues, we covered the water tank with a white box (0.6 m × 0.6 m × 1 m), which was rotated together with the water tank. After this manipulation, the cell activity was still tuned to the edges of the arena (Supplementary Figure S2b). In addition, a stability test of edge encoding cells indicated no significant difference (shuffle test) between the edge activity layer in the first and second half of the same recording session (Supplementary Figure S2c).

Next, we tested whether there was egocentric tuning to the walls^38–40^ by comparing the firing rate of the cells when fish was swimming with the edge to its right and the firing rate when the fish was swimming with the edge on its left. We defined the laterality index as the ratio between the difference in firing rate between the two sides to the sum of firing rates (see schematics Supplementary Figure S2e and “Methods”). According to this definition, egocentric edge cell has a laterality index of one, and none-egocentric cell has a laterality index of zero. We found that 12 out of the 15 edge cells had no significant tuning to the direction of the walls relative to the fish (Supplementary Figure S2f).

Population analysis showed that 11 out of 132; i.e., about 8% of all the well-isolated units recorded in 20 fish show edge-encoding properties. The edge activity layer of the cells ranged from 3.8 to 13.6 cm, with a mean distance of 9.2 cm and a standard deviation of 2.8 cm. The maximal firing rate of the cells near the walls ranged from 0.04 to 4.7 Hz with a mean of 0.7 Hz and a standard deviation of 1.35 Hz. In addition, the spike triggered average analysis identified 14 cells as having edge encoding properties.

**Figure 3:**
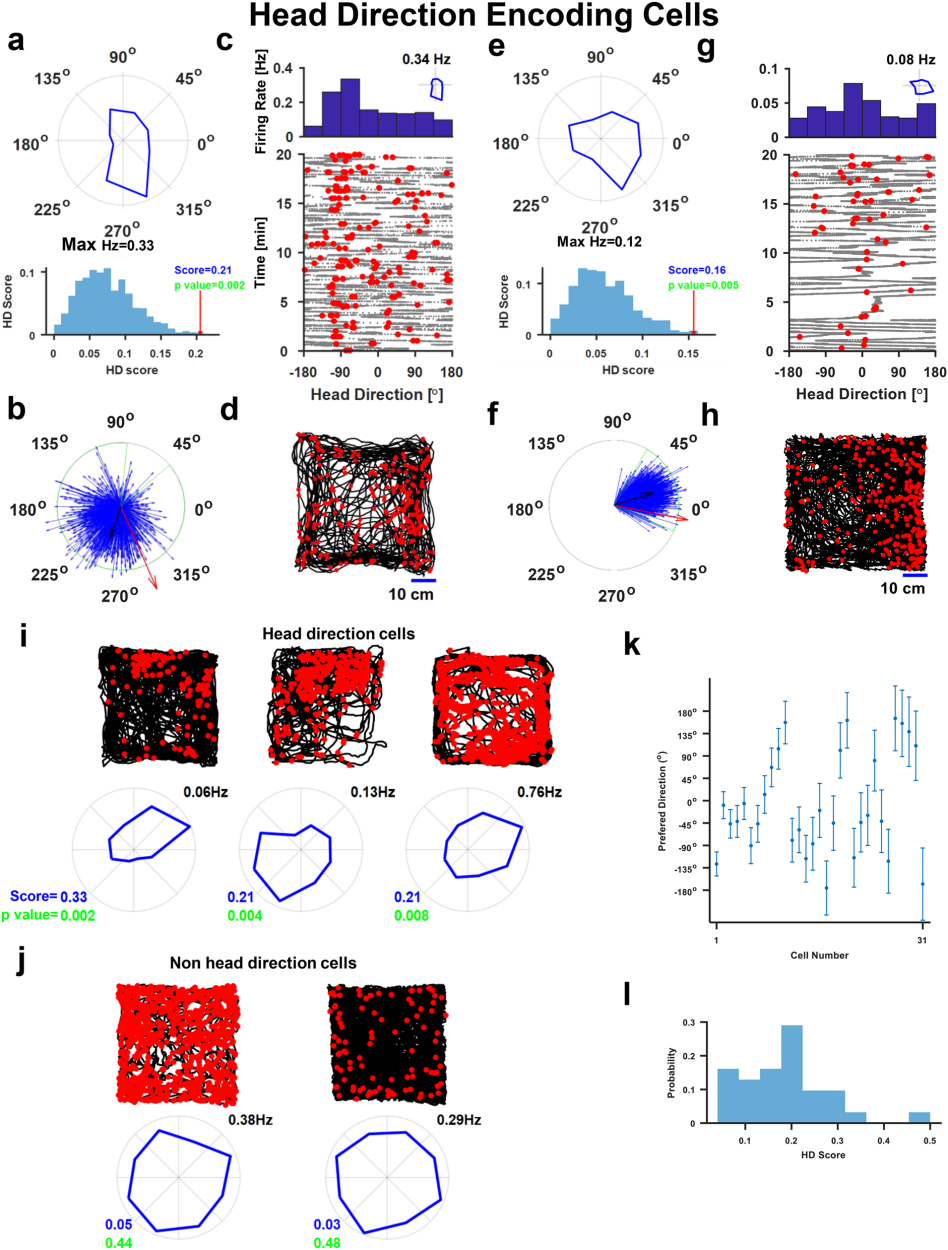
Head direction encoding cells in the goldfish lateral pallium. (**a**) Top Panel. Average firing rate as a function of head direction (maximal average firing rate is 0.33 Hz). The cellular response is tuned to the negative y-direction. Bottom panel**.** Statistical analysis; the cell's head direction score (red arrow) compared to the scores of 5,000 shuffled spike trains (blue histogram). The comparison of the red arrow and the histogram thus represents the statistical significance of the result. (**b**) Spike triggered average of the fish's head direction (red arrow) superimposed on 1,000 shuffled data examples (blue arrows). The black arrow represents the average of the 1,000 shuffled data examples and the green arc the 95% of shuffled data examples. (**c**) The first 20 min in (**a**) shows a stable representation. Top panel: firing rate as a function of head direction**.** Lower panel: fish head direction vs. time (grey dots) and spiking activity (red dots). (**d**) Scatter plot of the fish's positions when a spike occurred (red dots) superimposed on the fish trajectory (black curve). (**e**–**h**) An additional example of head direction cell with a full analysis as in (**a**–**d**). (**i**) Additional examples of head direction cells. (**j**) Two examples of non-head direction cells. (**k**) In-session preferred direction stability: mean values and standard deviation of the preferred direction of all cells (segment size of 7 min). (**l**) Head direction scores for all head direction cells (N = 31).

Head direction encoding cells are cells which generates a higher firing rate in the preferred orientation (Fig. 3). Examination of the average firing rate as a function of head direction revealed the directional tuning of head direction cells (Fig. 3a, see also the mean tuning curve of shuffled data and the histogram of all spike orientation in Supplementary Figure S3b,c). More examples are presented in Fig. 3e,i and in Supplementary Figure S3a; the quasi-isotropic directional tuning of other non-head direction cells presented in Fig. 3j. To assess the directional tuning of the cells, we used the length of the tuning curve mean vector (Rayleigh vector) as the head direction score, as standard in the field^41,42^. Then, we employed ISI shuffling procedure (see “Methods”) to obtain shuffled spike trains and measured the head direction index (also referred to as the score) of each of the shuffled spike trains to obtain a confidence interval. Cells whose head direction index was significantly larger than the shuffled data (p < 0.0125) were classified as head direction encoding cells (Fig. 3a,e, bottom panel, see “Methods”, see Supplementary Table ST1 for all encoding schemes of these cells). The head direction scores of the 31 head direction encoding cells ranged from 0.06 to 0.5 (mean 0.2, Standard deviation 0.1, Fig. 3i). In addition, we calculated the spike triggered average of the vector of the fish orientation and compared it to the average vectors of shuffled spike trains (see “Methods”). We found that the spike triggered average vectors of head direction encoding cells is significantly different from the shuffled examples (Fig. 3b,f).

We also tested the stability of the tuning curve across the entire experiment for all the head direction-encoding cells. This was done by dividing each experiment into segments and calculating the tuning within each segment. We found that the variability of tuning across segments was low (see “Methods”, Standard deviation of 30°–70°, and bin size 45° Fig. 3k). Furthermore, we found no specific bias in the distribution of the preferred orientation (Fig. 3k).

In total, population analysis identified that 31 out of 132 well-isolated cells showed head direction tuning using the Rayleigh vector analysis, i.e., 23% of all cells recorded in 20 fish. Their peak firing rates ranged from 0.13 to 3.6 Hz with a mean of 2.8 Hz and a standard deviation of 1.16 Hz. The spike triggered average analysis identified 23 cells as head direction encoding.

To test the stability of directional tuning across trials and to determine whether the head direction was tuned to the specificity of the visual scene, we rotated the water tank walls together with the painted visual cues by 180° between two recording sessions of a head direction encoding cell and masked the distant cues as before. After the rotation, the preferred direction of the cell rotated by ~ 130° (Supplementary Figure S3d). This is an indication that the cell was directionally tuned across different environments and that the tuning was dependent on the external visual cues and not on internal cues alone.

**Figure 4:**
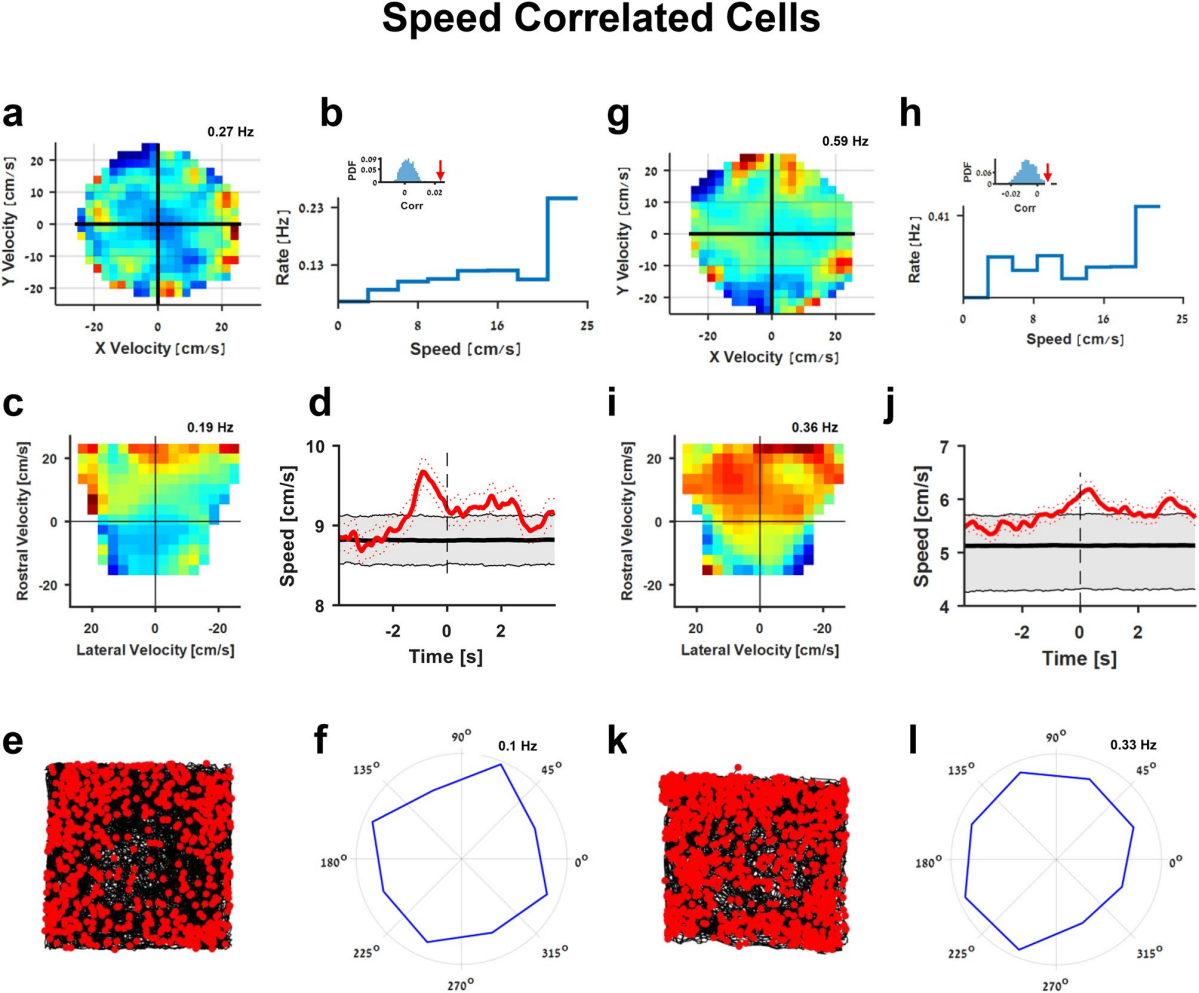
Speed correlated cells in the goldfish lateral pallium. (**a**–**f**) An example of a speed correlated cell. (**a**) Firing rate of the cell as a function of speed and direction in the velocity plane. The cell’s tuning to speed is manifested by the fact that the firing rate at the axis origin was low compared to the boundaries of the circle. (**b**) Firing rates of the cell in (**a**) as a function of the fish's speed the firing rate increased with the fish swimming speed. Insets show the correlation coefficient between the firing rate and the swimming speed for the cell in (**a**) (red arrow) vs. the correlation coefficients of the firing rate and the swimming speed of 5,000 shuffled spike trains. (**c**) The firing rate of the cell as a function of the velocity in the egocentric plane (rostral-lateral plane) shows that the cell is tuned to swimming in the rostral direction. (**d**) Spike triggered average of the fish's speed (red) superimposed on the 95% of shuffled data spike triggered average. (**e**) The fish's trajectory (black curve) and action potentials (red dots) recorded in the cell in (**a**). (**f**) Firing rate as a function of head direction of the cell in (**a**). The low score and quasi-isotropic shape suggest speed cells are independent of directional preferences. (**g**–**l**) An additional example of a speed cell (additional examples are presented in Supplementary Figure S4).

In addition to the edge and head direction encoding properties, we also found speed encoding in the goldfish lateral pallium. Speed encoding cells were correlated with the absolute value of the fish's swimming velocity. Two examples of speed encoding cells are presented in Fig. 4. The normalized firing rate map in the velocity-vector space (Fig. 4a,g, see “Methods”) revealed a pattern showing a tuning to speed, where the firing rate increased with speed regardless of the direction of motion (Fig. 4b,h). The normalized firing rate map in the velocity–vector egocentric reference frame (forward, backward, left, right) reveals that these cells were active mainly during the time the fish was swimming forwards (Fig. 4c,i). The deviations from a uniform distribution overall swimming direction result from the low firing rate.

The correlation coefficient between the neuronal firing rate and fish's speed was calculated, and ISI shuffling procedure (see “Methods”) was used to test for the speed selectivity of the neurons (see “Methods”) as standard in the field. The correlation coefficient between firing rate and speed showed a statistically significant pattern (insets, Fig. 4b,h). In addition, we calculated the spike triggered average of the fish swimming speed and compared it to the spike triggered average of shuffled spike trains (see “Methods”) in order to assess the speed encoding of the cells (Fig. 4d,j). Speed encoding cells had a significantly higher spike triggered average than shuffled data (p value < 0.0125, see Supplementary Table ST1 for all encoding schemes of these cells). We found that the spike triggered average analysis identified more cells as speed encoding cells than the correlation analysis (64 vs. 43). Additional examples of speed cells are presented in Supplementary Figure S4.

The population correlation coefficients of the speed cells were in the range of 0.008–0.065 (mean 0.028, standard deviation 0.016). The distribution of maximal speed ranged from 6 to 24 cm/s, with mean 11 cm/s and standard deviation 5 cm/s. Peak firing rates ranged from 0.1 to 4.4 Hz, with mean 0.5 Hz and standard deviation 0.8 Hz, and speed tuning curve slopes ranged from 0.005 to 0.25 Hz/cm/s, with mean 0.035 Hz/cm/s and standard deviation 0.05 Hz/cm/s. Finally, population analysis indicated that 43 out of 132; i.e., 33% of all well-isolated units recorded in the 20 fish could be classified as speed cells. The spike triggered average analysis identified 64 cells as speed encoding cells.

**Figure 5:**
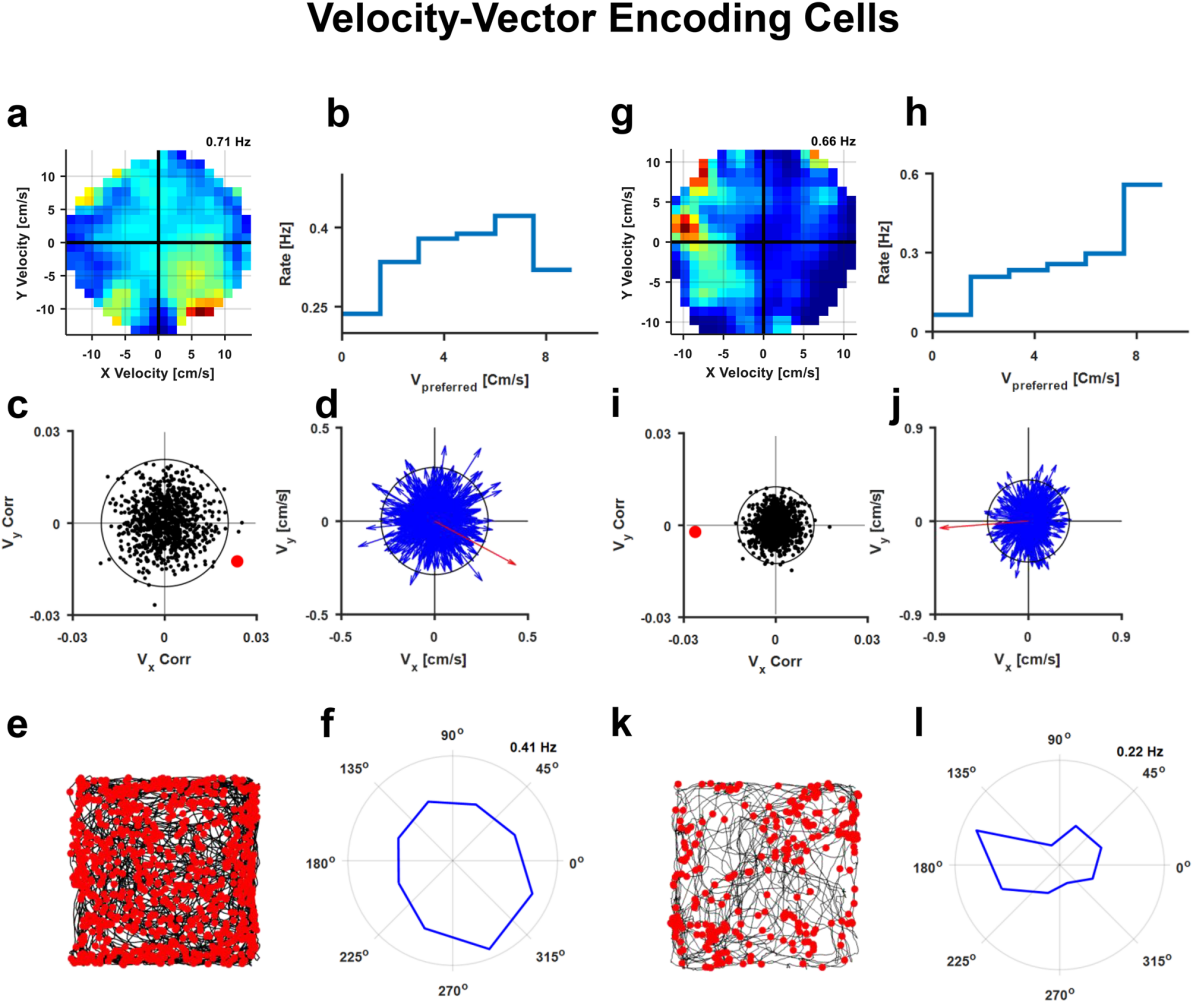
Velocity-vector cells in the goldfish lateral pallium. (**a**) Firing rate as a function of speed and direction in the velocity plane. (**b**) Firing rate as a function of velocity in the preferred directions (indicated as V_preferred_) of the cell in (**a**). The firing rate increased with the velocity-vector. (**c**) Two-dimensional correlations between firing rate and fish velocity-vector of the cell in a (red dot), compared to those of 5,000 shuffled spike trains (black dots). Black circle marks the 95th percentile of the shuffled correlations. (**d**) Spike triggered average of the velocity-vector of the cell (red arrow) compared with 1,000 examples of shuffled data (blue arrows). (**e**) The fish's trajectory (black curve) and action potentials (red dots) as recorded in the cell in (**a**). (**f**) Firing rate as a function of head direction of the cell in (**a**). The preferred direction corresponds to the preferred swimming direction shown in (**a**). (**g**–**l**) Another example of a velocity-vector encoding cell.

Conjunction of head direction and speed encoding were found in the form of cells that were active when the fish swam at high speed towards a specific direction regardless of its position; i.e., these cells correlate with the fish's velocity–vector rather than speed alone. Hence, these cells represent locomotion in space in an allocentric coordinate system, i.e., a world-centered reference frame. Examples of velocity-vector encoding cells are presented in Fig. 5. The normalized firing rate map in the velocity space (Fig. 5a,g) showed that cellular activity was correlated with both direction and speed. This corresponded to the firing rate as a function of speed in the preferred direction (Fig. 5b,h), and the patterns emerged from the head direction preference of these cells (Fig. 5f,l).

To test statistically whether a neuron was a velocity-vector encoding cell, we employed ISI shuffling procedure (see “Methods”), and compared the two-dimensional correlation between the firing rate and the velocity-vector of the recorded cell with the corresponding correlations of the shuffled neurons (Fig. 5c,i, p < 0.0125, see “Methods”). In addition to the two-dimensional correlation, we calculated the spike triggered average of the fish velocity-vector and compared it to the spike triggered average velocity-vector of shuffled neurons (Fig. 5d,j). The spike triggered average analysis identified more cells as vector-encoding cells than the correlation analysis (15 vs. 19). Additional examples of velocity-vector cells are presented in Supplementary Figure S5; all encoding schemes of the cells in Fig. 5 are presented in Supplementary Table ST1.

To test the velocity-vector tuning across recording sessions, we performed two control experiments. First, we rotated the water tank walls together with the painted visual cues by 180° between two recording sessions of a velocity-vector cell. After the rotation, the preferred direction of the cell rotated by ~ 150° (Supplementary Figure S6a). In the second control, we transferred the fish from a square water tank to a circular one, while recording the same velocity cell. In both sessions, the cell showed velocity-vector encoding, and the preferred direction changed in 80° (Supplementary Figure S6b). The change in directional preference between different environments suggests that when moving to a new environment, the fish forms a new coordinate system in the absence of absolute directions such as in a compass. A stability test of velocity-vector encoding cell indicated no significant difference (shuffle test) between the first and second half of the same recording session (Supplementary Figure S6c).

In total, 15 out of 132; i.e., 11% of all units recorded in 20 fish had velocity-vector encoding properties. The distribution of maximal speed ranged from 8 to 25 cm/s, (mean 14 cm/s and standard deviation 5 cm/s), peak firing rates ranged from 0.02 to 3.3 Hz (mean 0.5 Hz and standard deviation 0.8 Hz). Velocity-vector correlation to spiking activity ranged from 0.01 to 0.03 (mean 0.02 and standard deviation 0.007). In addition, the spike triggered average analysis identified 19 cells as velocity vector encoding cells.

**Figure 6:**
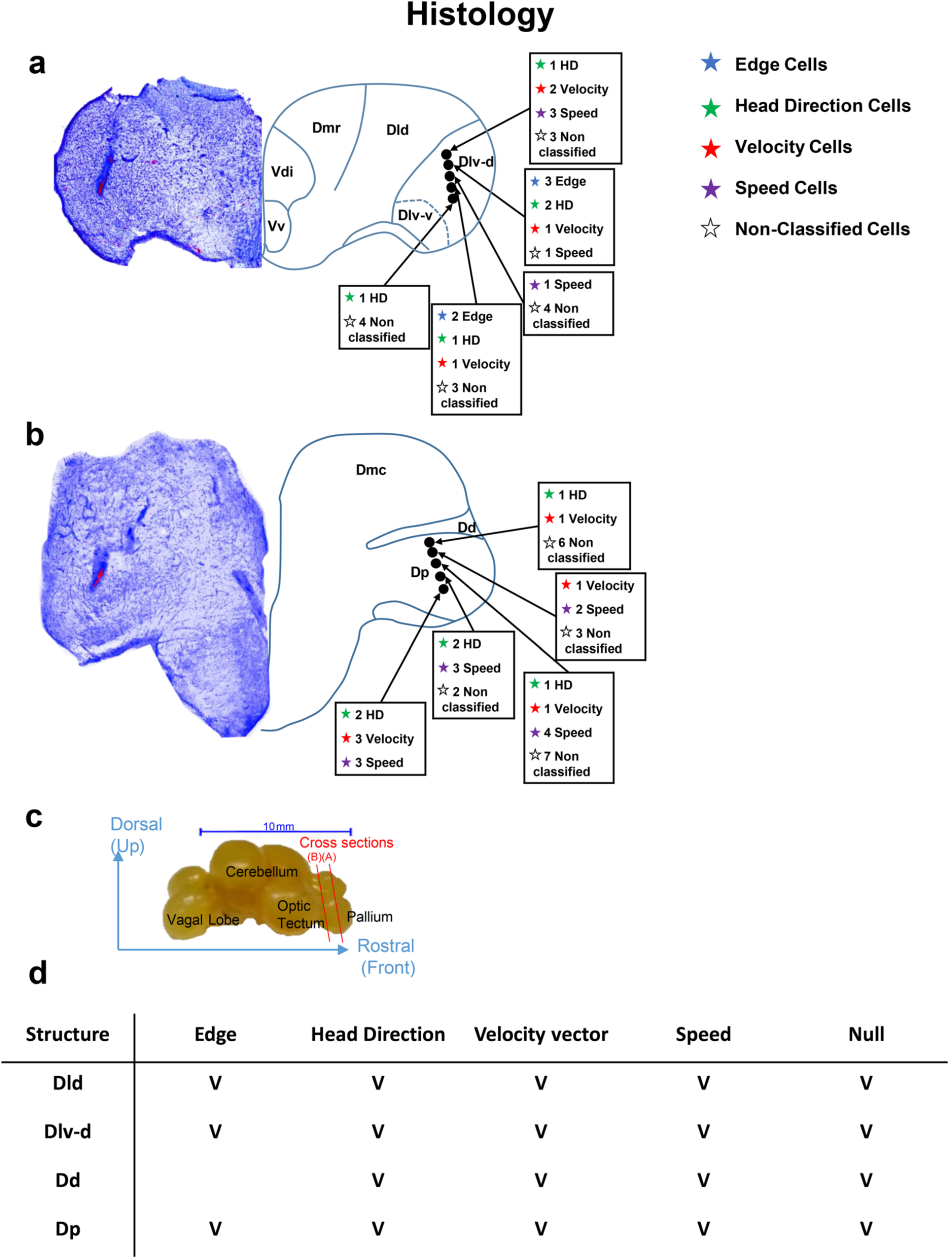
Distribution of cells in the goldfish brain. (**a**,**b**) Two examples of recording locations and cell encoding types found in those locations (anatomical diagram based on Northcutt^45^). (**c**) Fish brain structures. Red cross sections a and b are in correspondence to the brain sections presented in (**a**) and (**b**), respectively. (**d**) Summary of the recording locations in the brains of 11 fish and the encoding schemes found in these locations.

After the recording, we located the recording site in eleven of the fish using standard post-recording anatomical procedures. The different encoding schemes were found mainly in lateral areas of the fish pallium^43–45^ (Fig. 6, more details in Supplementary Figure S7, see “Methods”). Overall, we found that 72 out of 132; i.e., 55% of all units recorded in 20 fish had at least one encoding property. Out of those 72 cells, 72% showed tuning to only one encoding scheme, and 28% showed mixed tuning.

## Discussion

We found edge, head direction, velocity-vector, and speed encoding properties in the activity of cells in the goldfish lateral pallium. Thus, these cells represent different components of position and locomotion in the goldfish brain. The founding of these encoding properties in the lateral structures of the goldfish pallium suggests that these regions contain a variety of neurons that represent features of space, direction, and speed.

The activity of edge encoding cells in the goldfish pallium is tuned to the edges of the arena, with no significant preferences to a specific edge, unlike mammalian border cells^46^. These cells might provide the reference frame for different navigational strategies. The fish head direction encoding cells were shown to have stable tuning to the fish's orientation and represented a sparse directional signal. These cells differ in two main ways from the mammalian head direction cells^47^: the firing rate is sparse, and there is some activity in the null direction. The fish's speed correlated cells exhibited much lower firing rates than the speed cells found in the medial entorhinal cortex^16^. Finally, the velocity-vector cells were found to be a conjunction of head direction and speed encoding.

These findings, together with finding from previous lesion studies^48,49^, hint that these cells may constitute the basic building blocks of the goldfish navigation system and can thus help to evaluate theories of biological navigation systems derived from observations of the mammalian system alone. Further investigations are needed to obtain a complete functional map of this region and to understand whether it plays a foundational role in the neural navigation system of the teleost brain.

In the mammalian system, there are additional two principal cell types, which represent space and are part of the navigation system, place cells, and grid cells. As for now, we did not find any cell with an apparent spatial tuning in the form of place or grid cells. The lack of these cell types in our recordings does not negate the existence of these cell types in other brain areas or a low percentage (< 0.7%). A previous method paper by Canfield and Mizumori has shown preliminary evidence of one spatially tuned cell in the goldfish lateral pallium^32^. Their method is based on a commutator, which might restrict the swimming of the fish as it seems that the fish was mainly swimming near edges. They report on a single spatially tuned cell and another cell, which fires when the fish is inside a clay pot, however, the fish did not cover the entire water tank area. In another study, Fotowat et al. have looked for spatial tuning in the weekly electric fish DDi region of the telencephalon, which is interconnected to the Dlv^50^. They assessed the place specificity of 21 single cells using spatial information alone. They found eight units with significant spatial information. As in the Canfield and Mizumori report, most of the fish were mainly swimming near either edges or objects, and the firing of the units increased near the objects. In our study, we did not use the Shannon Information measure in order to classify the space specificity of our findings since such information measure is biased for low firing rate as observed in the goldfish.

Another critical component of the spatial representation in the rodent hippocampal formation is theta oscillations, the rodent place cells are locked to the theta phase, and their specific phase represents the location inside their place field. These oscillations do not exist in all mammalian hippocampal formation, and place cells can exist without them^51,52^. In fish, very little is known about brain oscillations, and nothing is known about theta oscillation in the telencephalon. Future studies will assess the existence of place cells in the goldfish pallium in different areas and under different spatial tasks and the connection between our findings and the fish brain oscillations.

Finding edge, direction, and speed tuned cells in the fish's lateral pallium, as found in the mammalian hippocampal formation, suggests that the representation of edges or borders and self-kinematics may have evolved from an ancient neural circuit common to both teleost and mammals. Therefore, these results have implications for current theories on the evolution of brain function in the teleost and outside the teleost lineage. They suggest the lateral pallium as the functional homologue of the hippocampal formation, which consists of several sub-regions in the mammalian brain, including the hippocampus, the entorhinal cortex, the subiculum and the post-subiculum^53^. Currently, however, it is impossible to determine the exact homology between the divisions of the mammalian hippocampal formation and the divisions of the pallium in the teleost brain^53^.

Although there is a robust consensus as to the inventory of cells that represent space in the mammalian brain^5,7,10^, there is no accepted theory based on empirical data as to how these spatial representation components are integrated in the brain into a functioning navigation system^5^. One solution may emerge from studying the neural mechanisms underlying navigation in other vertebrate lineages, since elucidating the mechanisms of space representation in different vertebrate taxa can help decipher how the elementary building blocks of navigation were integrated throughout evolution into a functional navigation system^51,52,54–56^. Crucially, comparative studies can help determine whether the mammalian navigation system is unique or an instance of a more general biological design. Thus, a comparative approach may help resolve the key question of the critical components making up a functioning navigation system.

In our recordings, we found that the spiking activity in the fish pallium was rather sparse, and lacked the characteristic spike bursts found in certain loci in the mammalian hippocampal formation^57^. However, the firing rates we observed were similar to the ones observed in recordings from the behaving weakly electric fish lateral pallium^50^ and recordings from brain slices of goldfish pallium and slices of the weakly electric fish dorsal pallium^58^. These observations raise the question of the ways in which the fish brain controls its behavior using this sparse coding.

Only a small number of studies have attempted to study the neural representation of self-kinematics and space outside of the mammalian lineage. The Bingman et al.^59^ analysis of place cells in the pigeon's hippocampus homologue only found preliminary evidence of spatial encoding. Recently, Payne and Aronov reported the first evidence of place cells in the food-caching bird^60^. In addition, the Canfield and Mizumuri method paper describes an extracellular recording system in tethered goldfish and provides preliminary evidence for speed and place encoding, as discussed above^32^. Ahrens et al.^61^ studied the brain of paralyzed larval zebrafish during fictive navigation in virtual environments that contained dark and light areas. Although it is possible that the activity pattern they observed represents place features in the virtual environment, the authors suggest it is more likely that the activity patterns are tuned to the lighting conditions. A complementary study in the telencephalon of weakly electric fish reported supporting evidence for cells that are more active near objects and edges as discussed above^50^. Contrary to the results reported here, the authors also described speed correlated cells, which are only sensitive to a sudden increase in the fish's speed (Fig. 4d,j). Finally, studies in the fruit fly have documented representations of orientation with respect to salient landmarks in the environment^8,62^.

Overall, this study constitutes a step toward a better understanding of the navigation system in non-mammalian vertebrates. This study establishes the basic inventory of spatial and kinematical cells in the goldfish lateral pallium. Future work will exploit the spatial and kinematical encoding in a full three-dimensional environments and the encoding during spatial tasks.

## Methods

### Experimental model and subject details

Goldfish (*Carassius auratus*), 13–15 cm in body length, 80–120 g body weight were used in this study. A total of 20 fish were used for the recordings. The fish were kept in a water tank at room temperature. The room was illuminated with artificial light on a 12/12 h day-night cycle. The fish were kept in the home water tank and were brought to the experimental water tank for recordings.

When fish are placed in an unfamiliar environment, they tend to stay near the walls or barely swim. To avoid this behavior, fish were first trained to explore the entire water tank. In the training sessions, the fish swam freely in the tank for 20 min a day for several days, while an automatic feeder positioned above the center of the tank fed the fish as soon as they approached the center of the tank. After about a week of training, most fish were familiar with the water tank and explored it efficiently.

All the experiments in goldfish were approved by the Ben-Gurion University of the Negev Institutional Animal Care and Use Committee and were in accordance with the government regulations of the State of Israel.

### Method details

#### Wireless electrophysiology

The behavioral fish electrophysiology is described in detail in Vinepinsky et al.^36^ and Cohen and Vinepinsky et al.^37^. Briefly, the experimental setup for recording extracellular signals from the brain of freely swimming goldfish operates through a small data logger (Mouselog-16, Deuteron Technologies Ltd., Jerusalem, Israel). The logger is connected to an implant mounted on the fish's skull and receives input from one or two tetrodes placed in the fish brain. The implant contained a microdrive, which allowed us to move the tetrodes between recording sessions. In addition, we place a reference electrode near the fish brain for the detection of possible motion artifacts. To protect the electronics, the neural logger is placed in a waterproof case (Fig. 1A). The data logger is controlled wirelessly by a computer via a transceiver (Deuteron Technologies Ltd., Israel) and records the neural signals at 31,250 Hz using a 300 Hz high-pass analog filter. A Styrofoam marker is mounted on the waterproof case for the entire implant (tetrodes, box, logger, and battery) to be buoyancy neutral (i.e., total average density of 1 g/cm^3^). The front and back ends of the Styrofoam marker are painted in different colors to determine the swimming direction from the video recordings easily.

To ensure that our recordings were free of motion artifacts, we performed a control experiment where we moved the fish in the water tank and bumped them on the foam sheet walls while recording neural activity (see example in Supplementary Figure S1i–j). This was done at the end of each surgery, while the fish was still under anesthesia. Only trials devoid of any motion artifact were used for further analysis.

#### Surgery and stereotaxic procedure

Surgery was done out of the water while the fish was anesthetized and perfused through its mouth (MS-222 200 mg/l, NaHCO3 400 mg/l 1, Cat A-5040, and Cat S-5761, Sigma-Aldrich, USA) as described in Vinepinsky et al.^36^. The brain location was targeted by constituting the anterior mid margin of the posterior commissure as the zero point for the stereotaxic procedure as described by Peter and Gil^63^. From the zero point, using a mechanical manipulator, we moved the tetrodes 1 mm laterally, 1 mm anteriorly and 1.5 mm ventrally. Using the built-in microdrive, we were able to move the tetrode in the dorsal/ventral axis in between sessions.

#### Water tank

The water tank for the experiment was 0.6 m × 0.6 m × 0.2 m in size and was coated with a foam sheet (Supplementary Fig. S8). Visual landmarks were marked on the walls of the foam sheets under the water level. A circular arena with a radius of 0.34 m and a height of 0.2 m was used for some of the experiments (Supplementary Figures S4 and S5).

#### Video recording

A camera above the water tank was used to localize the fish in the X–Y plane. All sessions were recorded using a "Gopro hero 4" camera at 24 FPS, H.D. resolution, and a linear field of view. To synchronize the neural activity and the video recordings, we used the synchronization system provided with the Mouselog-16 by Deuteron Technologies. We set the Mouselog-16 transceiver to deliver a 100 ms wide pulse at intervals of 10 and 20 s. The signal was then sent to the camera's audio input.

#### Recording sessions

Each recording session involved synchronized recordings from the Neurolog-16 and the camera system while the fish navigated freely in the water tank. Recording sessions lasted about 1 h. To avoid the issue of whether cells recorded from the same location in different sessions are identical, we included in the analysis only data from different locations in different sessions (See Supplementary Table ST2 for complete details).

#### Histology

The brains of eleven of the fish were fixed in 4% paraformaldehyde overnight (Electron Microscopy Sciences, CAS #30525-89-4), then immersed in a 40% glucose solution for cryoprotection. After freezing, the brains were cryo-sliced (40 μm slices), and Nissel stained to reveal the electrode position in the brain (i.e., the lateral pallium or any other brain area targeted for recording).

### Quantification and statistical analysis

All analyses were conducted using an in-house Matlab program.

#### Data synchronization

The camera's timing was adjusted to the Mouselog-16 timing using the synchronization pulses recorded in the audio channel.

#### Spike sorting

Offline, the raw data were filtered using a bandpass filter of 300–7,000 Hz. Then, action potential timings were detected using a threshold detector. Subsequently, standard spike sorting was done by manual clustering using PCA analysis of the spike amplitudes, widths, and waveforms across all the electrodes in each tetrode^64,65^. To ensure that our result does not depend on spike sorting errors, we have done the spike sorting in the following way. Two individuals analyzed each dataset separately. Only units that were separated in the PCA space were ranked well enough for further analysis. All units that were not separated in the PCA space were discarded. In addition, for each manually cut cluster, we have tested different cluster borders and ensure that the results do not depend critically on specific borders. All units that showed instability were also removed from further analysis. Finally, spike waveforms that were present simultaneously in the two recording tetrodes or the reference electrode were removed from the analysis (see examples in Fig. 1b–e and Supplementary Figure S1a–h).

#### Fish trajectory and firing rate map analysis

The fish's location and orientation in each video frame were detected using the colored styrofoam marker that was attached to the case containing the logger. The water tank was tessellated to 5 cm^2^ bins and smoothed using a 2-dimensional Gaussian (sigma = 7.5 cm). This yielded two auxiliary maps, which indicated how many spikes occurred in each bin and how much time the fish spent in each bin. The firing rate map for each neuron was obtained by dividing the two maps bin by bin, i.e., occupancy corrected. Bins that were not visited enough by the fish were discarded from the analysis and appear on the map as white bins. Sessions in which the fish only swam around the edges were discarded.

#### Multiple comparisons correction

We have analyzed and tested the cells for the encoding of four different properties: edge distance, head direction, speed, and velocity-vector. When multiple statistical tests are used on the same data, there is an increase in observing rare events in one of the tests. To correct for this, we used the Bonferroni correction and set the p-value for each test to be 0.0125 to get a total p-value of 0.05 for the multiple comparisons.

#### Shuffling procedure

In all of the following analyses, we have used inter-spike intervals (ISI) shuffling procedure to create shuffled spike trains. Each shuffled spike train was obtained by first calculating the ISI, shuffling it by random permutation, and using the cumulative sum to obtain the shuffled spike train. This shuffling procedure kept the number of spikes and ISI histogram the same.

#### Edge encoding analysis

To determine which cells showed edge encoding properties, we measured the width of the edge activity layer near the walls of the water tank that contained 75% of the spikes. We compared this to 5,000 shuffled spike trains and calculated the probability of observing a width of edge activity layer by comparing the value obtained for the recorded cell to the values calculated for the shuffled spike trains. Cells with a p-value below 0.0125 were considered to be edge encoding (Fig. 2d,i,n). Sensitivity analysis of the edge activity layer definition was done by calculating activity layers that contained 0–100% of the spikes (Fig. 2c,h,m).

#### Spike triggered averaged edge distance

To further ensure the edge encoding properties of the cells, we calculated the spike triggered average (STA) of fish distance from the water tank walls. This was done by calculating the fish distance from the wall during the 4 s before and after a spike has occurred, then summing the distances over all spikes and dividing by the number of spikes. Next, we calculate the spike triggered average fish distance of 1,000 shuffled spike trains using the shuffling procedure described above. Cells in which the spike triggered average distance was lower than the 98.75 percentile (p-value < 0.0125) of the shuffled spike triggered average was considered significant.

#### Egocentric edge tuning analysis

In order to assess the edge cell tuning to the wall's egocentric direction (i.e., left or right), we first calculated the egocentric position of the nearest wall over the entire fish trajectory. We took into account only positions where the fish was within 10 cm from the wall. Then we calculated the firing rate of the cell while the fish was swimming with the edge to its left (± 60°) and the firing rate of the cell while the fish was swimming with the edge to its right (see supplementary Figure S2e,f). We defined a laterality index as $abs\left( {\frac{{{\text{FiringRate}}_{{{\text{Left}}}} {-}{\text{FiringRate}}_{{{\text{Right}}}} }}{{{\text{FiringRate}}_{{{\text{Left}}}} + {\text{FiringRate}}_{{{\text{Right}}}} }}} \right)$. The laterality index is defined to be one when all spikes occurred when the wall was on one side of the fish, i.e., the edge encoding cell is egocentric, and zeros when there is no egocentric encoding effect. Next, we simulated 5,000 edge cells by shuffling the spike timing while keeping the distribution of the spikes distance from the wall fixed. In that way, the shuffled spike train conserved the edge tuning. Finally, we compared the laterality index of the original edge cell to the laterality index of the simulated edge cells. We defined the p-value as the fraction of simulated edge cells with a higher directionality index than the original cell. Cells with p-value < 0.0125 were defined as having significant egocentric tuning.

#### Head direction tuning

The head direction tuning was computed using 45° bins. For each bin, we have counted how many spikes occurred while the fish orientation was in this bin, then, for occupancy correction, we divided this number by the total time the fish spent in that orientation, therefore got the average firing rate of this bin in Hz.

#### Head direction score

The head direction score was defined as the mean Rayleigh vector length on the tuning curve, as is standard practice, using 45° bins^41^. For classification, we calculated the length of the Rayleigh vector for 5,000 shuffled spike trains and compared it to the length of the cell's Rayleigh vector. Cells were defined as head direction cells if their score was significantly higher than the shuffled scores (p < 0.0125).

In-session directional tuning standard deviation was calculated by splitting each session into 7-min segments and calculating the preferred direction in each segment (Fig. 3g).

#### Spike triggered averaged head direction vector

In order to calculate the spike triggered averaged (STA) head direction vector, we first identified the fish orientations during all spike times and then calculated the mean vector of these angles. Next, we calculate the STA head direction vector of 1,000 shuffled spike trains using the shuffling procedure described above. Cells with a mean vector longer than the 99.375 percentile of the shuffled data vector length or that their mean vector angle was not inside the arc that contained 99.375% of the shuffled data mean vector angles were considered to have a significant spike triggered average (p-value < 0.0125).

#### Firing rate map in the velocity plane

The fish's two-dimensional velocity-vector was tessellated to using 100 bins, ten in each dimension, and smoothed using a 2-dimensional Gaussian. This yielded two auxiliary maps, which indicated how many spikes occurred in each bin and how much time the fish spent in each bin. The firing rate map for each neuron was obtained by dividing the two maps bin by bin, i.e., occupancy corrected. Bins that were not visited enough by the fish were discarded from the analysis and appear on the map as white bins.

#### Speed correlation analysis

To classify speed cells, we calculated the correlation coefficient between the cellular firing rate and the speed of the fish, as is commonly done^66^. Then, 5,000 shuffled spike trains were obtained by first calculating the inter-spike intervals, shuffling the inter-spike intervals by random permutation, and using the cumulative sum to obtain the shuffled spike train. For each shuffled spike train, the correlation coefficient between the firing rate and swimming speed was calculated. Comparing the result of the shuffled spike trains to the cellular result yielded an estimate of the p-value of each cell. The cell was defined as a speed cell if p < 0.0125. Cells with an overall firing rate of less than 0.05 Hz were omitted from this analysis due to the low statistical significance.

#### Spike triggered averaged speed

To ensure the speed encoding properties of the cells, we calculated the spike triggered average (STA) swimming speed. This was done by calculating the fish speed during the 4 s before and 4 s after a spike has occurred, then summing the swimming speed over all spikes and dividing by the number of spikes. Next, we calculated the spike triggered average of fish speed of 1,000 shuffled spike trains using the shuffling procedure described above. Cells in which the spike triggered averaged speed was higher than the 98.75 percentile (p-value < 0.0125) of the shuffled STA was considered significant.

#### Velocity-vector cell analysis

To classify the velocity-vector cells, we calculated the correlation coefficient between the cellular firing rate and the velocity-vector of the fish. Then, 5,000 shuffled spike trains were obtained by first calculating the inter-spike intervals, shuffling the inter-spike intervals by random permutation, and using the cumulative sum to obtain a shuffled spike train. For each shuffled spike train, the correlation coefficient between the firing rate and fish's two-dimensional velocity-vector was calculated. By comparing the result of the shuffled spike trains to the cellular result, we obtained an estimate of the p-value of each cell. A cell was defined as a velocity-vector encoding cell when p < 0.0125.

#### Spike triggered averaged velocity-vector

To further ensure the velocity-vector encoding properties of the cells, we calculated the spike triggered average (STA) velocity-vector. This was done by calculating the fish velocity-vector during the times in which a spike has occurred, then summing the velocity-vectors over all spikes and dividing by the number of spikes. Next, we calculate the spike triggered average velocity-vector of 1,000 shuffled spike trains using the shuffling procedure described above. Cells in which the spike triggered average velocity-vector was longer than the 98.75 percentile (p-value < 0.0125) of the shuffled spike triggered average was considered significant.

#### Testing for weakly selective spatial tuning hypothesis

In order to test whether a population of weakly selective spatial cells shows the same edge, head direction, and speed encoding properties as our data set, we have simulated three types of data sets. A data set of non-spatial cells, a data set of weakly selective spatial cells with a Gaussian place field with a sigma of 30 cm, and a data set of weakly selective spatial cells with Gaussian place field with a sigma of 40 cm. Each simulated data set was constructed using the same trajectories as our original data set and with the same mean firing rates of cells. Then, we tested whether the simulated cell population has edge, head direction, speed, or velocity encoding. We repeated the process 100 times for each selection of weakly spatial tuning (i.e., 30 cm, 40 cm, or uniform). We have found that the simulated data set cannot reproduce our results (Supplementary Figure S9). To conclude, this analysis shows that a weakly spatially selective cell population cannot explain our results.

## Supplementary information

Supplementary Information.
