# Supplementary material for: Representation of edges, head direction, and swimming kinematics in the brain of freely-navigating fish
Source: Sci Rep. 2020 Sep 8;10:14762. doi: 10.1038/s41598-020-71217-1 (PMC7479115; doi:10.1038/s41598-020-71217-1)
Supplement: Supplementary file 1 — Supplementary Information. [file 41598_2020_71217_MOESM1_ESM.pdf]

# Representation of Edges, Head Direction, and Swimming Kinematics in the Brain of Freely-Navigating Fish

**Ehud Vinepinsky[1,2], Lear Cohen[2,3], Shay Perchik[2,4], Ohad Ben-Shahar[2,5], Opher Donchin[2,3] and Ronen Segev[1,2,3, *]**

[1]Department of Life Sciences, Ben Gurion University of the Negev, Beer Sheva 84105, Israel
[2]Zlotowski Center for Neuroscience, Ben Gurion University of the Negev, Beer Sheva 84105, Israel
[3]Department of Biomedical Engineering, Ben Gurion University of the Negev, Beer Sheva 84105, Israel
[4]Department of Cognitive and Brain Sciences, Ben-Gurion University of the Negev, Beer Sheva 84105, Israel
[5]Department of Computer Sciences, Ben Gurion University of the Negev, Beer Sheva 84105, Israel
[*]Correspondence: ronensgv@bgu.ac.il

## Supplementary Material

# Supplementary Table ST1 – Vinepinsky et al.

| Figure | Edge | Head Direction | Speed | Velocity |
|---|---|---|---|---|
| 2a-e | V | - | - | - |
| 2f-j | V | - | - | - |
| 2k-o | V | V | - | - |
| 3a-d | - | V | V | V |
| 3e-h | - | V | - | - |
| 3i left | - | V | - | V |
| 3i middle | - | V | - | - |
| 3i right | - | V | - | - |
| 3j left | - | - | V | - |
| 3j right | - | - | V | - |
| 4a-f | - | - | V | - |
| 4g-l | - | - | V | - |
| 5a-f | - | V | - | V |
| 5g-l | - | V | V | V |

# Supplementary Table ST2 – Vinepinsky et al.

| Fish | Day | Session | single units |
|---|---|---|---|
| 566 | 4 | 1 | 3 |
| 568 | 1 | 1 | 2 |
| 570 | 1 | 1 | 2 |
| 571 | 1 | 1 | 1 |
| 571 | 1 | 2 | 2 |
| 571 | 2 | 1 | 1 |
| 571 | 2 | 2 | 1 |
| 571 | 3 | 1 | 3 |
| 571 | 3 | 2 | 2 |
| 571 | 4 | 1 | 1 |
| 578 | 1 | 1 | 9 |
| 581 | 1 | 1 | 1 |
| 582 | 1 | 1 | 2 |
| 582 | 2 | 1 | 4 |
| 584 | 2 | 1 | 2 |
| 560 | 1 | 1 | 0 |
| 300 | 1 | 1 | 1 |
| 304 | 2 | 1 | 0 |
| 306 | 4 | 1 | 1 |
| 316 | 2 | 1 | 2 |
| 321 | 1 | 1 | 5 |
| 321 | 2 | 1 | 4 |
| 322 | 4 | 3 | 5 |
| 331 | 1 | 1 | 3 |
| 331 | 2 | 1 | 4 |
| 331 | 2 | 3 | 7 |
| 331 | 3 | 1 | 3 |
| 331 | 3 | 3 | 4 |
| 332 | 1 | 1 | 7 |
| 332 | 2 | 1 | 6 |
| 332 | 3 | 1 | 5 |
| 332 | 4 | 1 | 5 |
| 332 | 5 | 1 | 5 |
| 346 | 5 | 1 | 4 |
| 349 | 2 | 1 | 7 |
| 349 | 3 | 1 | 4 |
| 112 | 1 | 2 | 7 |
| 112 | 2 | 2 | 3 |
| 112 | 3 | 2 | 4 |

# Supplementary Figure S1 – Vinepinsky et al.
## Spike sorting examples

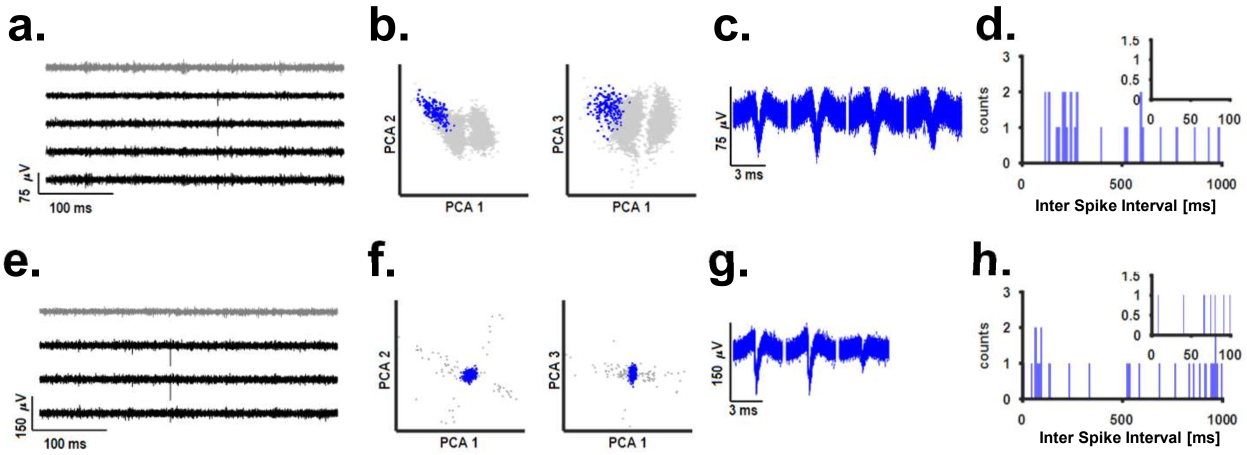


## Noise control experiment

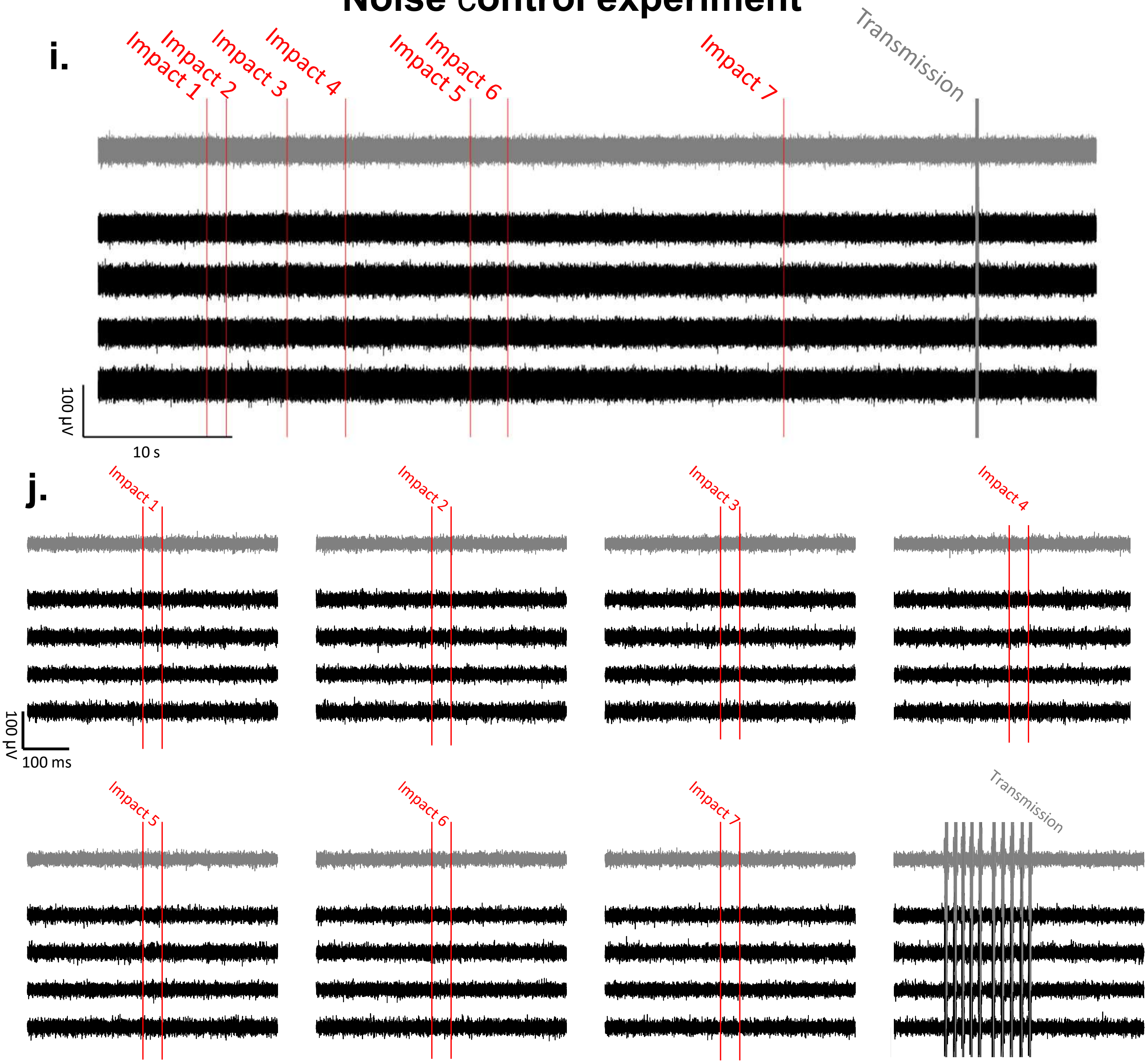

**Supplementary Figure 1| Additional Spike Sorting Examples. a** and **e.** Examples of raw recordings from a tetrode (black traces) and a reference electrode (gray) in the fish's lateral pallium. Neural activity is observed on the tetrode alone. **b** and **f**. Projection on the first three principal components of the data from the tetrodes in A and E, respectively, of all candidate spikes (gray dots) that crossed the threshold. Blue dots indicate a single cell cluster. In panel F only one main cluster crossed the detection threshold. **c** and **g.** Waveforms of two neurons (blue clusters from B and F, respectively) after spike sorting. **d** and **h**. Inter spike interval histogram of the detected clusters. Insets show there were no violations of the refractory period in either case. The presented clusters form a border cell (Figure 2A-D) and a velocity cell (Supplementary Figure 5A, left column), respectively. **Noise Control Experiment.** To guarantee the absence of spike-like artifacts in the recording system caused by fish motion, we conducted a control experiment where we generated impacts with the walls while the fish was anesthetized. This was done for all fish **i**. Raw recordings from a tetrode (black traces) and a reference electrode (gray) during a control experiment (same recording day and fish as in Figure 1). Impacts with the walls are marked by red lines, communication artifact is easily detected. Since the fish was under anesthesia, brain activity was suppressed. No motion or impact artifacts were present. **j.** Zooming in on **i**. The start and the end of each frame in which an impact occurred marked in red lines. No motion or impact artifacts were present. Communication artifact (bottom right panel) is easily detected since it affects all of the recording channels and has a unique signature. The logger communicates with the control computer every two minutes for about 150 ms to synchronize their clocks.

# Supplementary Figure S2 – Vinepinsky et al.
## **Edge encoding**

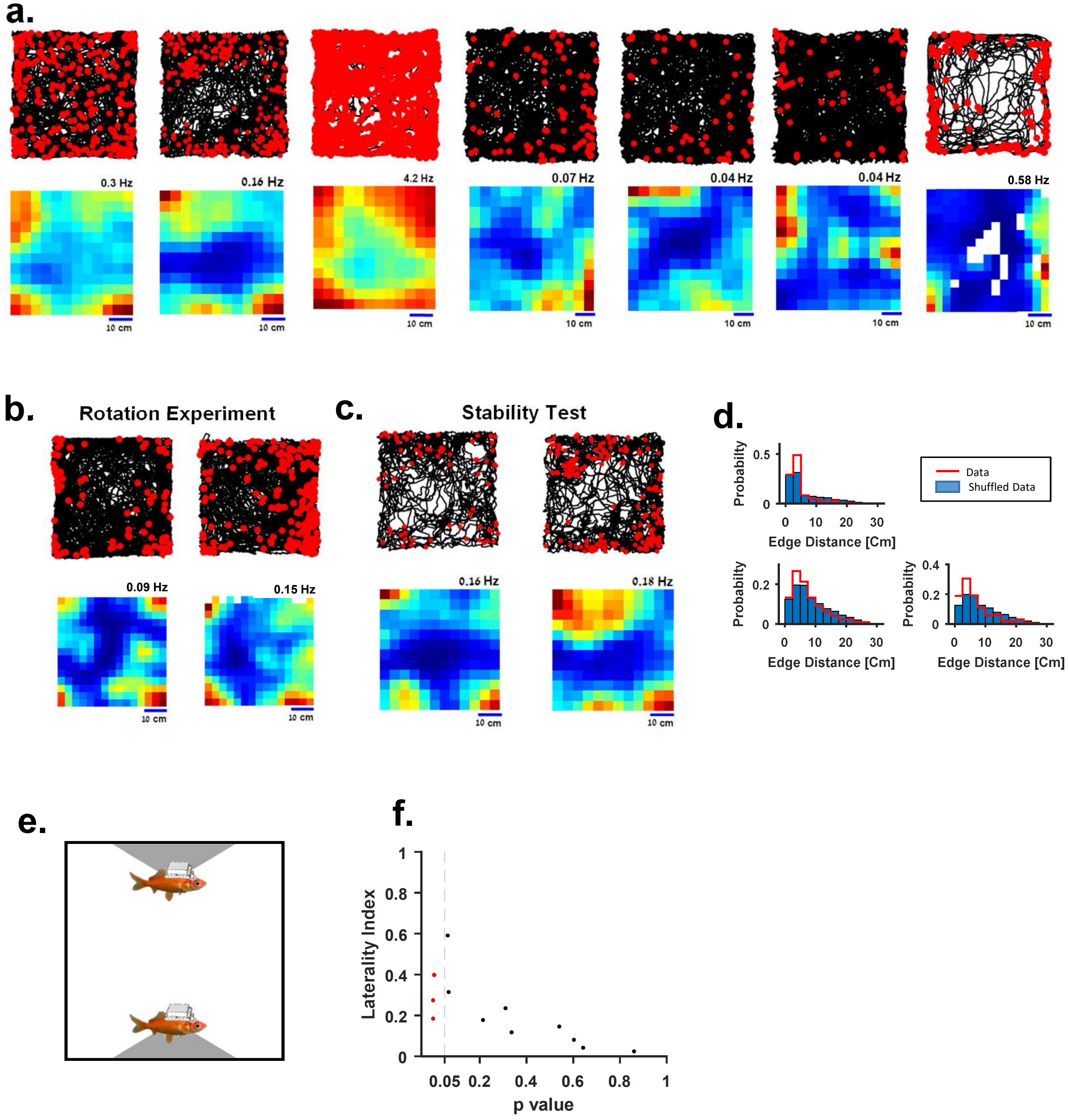

**Supplementary Figure 2| Edge Encoding Cells. a.** Additional examples of border cells. The cells were recorded in different sessions in different fish. Top panel, fish trajectory and action potentials (red dots). Bottom panel, firing rate heat map (color coded from dark blue, zero firing rate, to dark red, maximal firing rate, indicated in top right side of each panel). **b.** Visual cues rotation: two sequential sessions in which the visual cues were rotated in 180 degrees before the second session. Fish trajectories (top panels) and firing rate heat map (lower panels, as in a) are presented. Cell activity shows border proximity characteristics in both cases. Right column is the same cell as in Figure 2 k-o. **c.** Split session stability test: edge encoding cell (same as a, second column from left) activity in the first half (left panels) and the second half (right panels) of the session. Cell shows border proximity properties in both parts and the border activity layer did not change significantly (9.9 cm and 9.6 cm, permutation test). **d.** Comparison of the distribution of edge distances of the cells examples in Figure 2 and shuffled spike strains. The distributions of the cell edge distances is higher for shorter distances.

# Supplementary Figure S3 – Vinepinsky et al.
## **Head direction encoding**

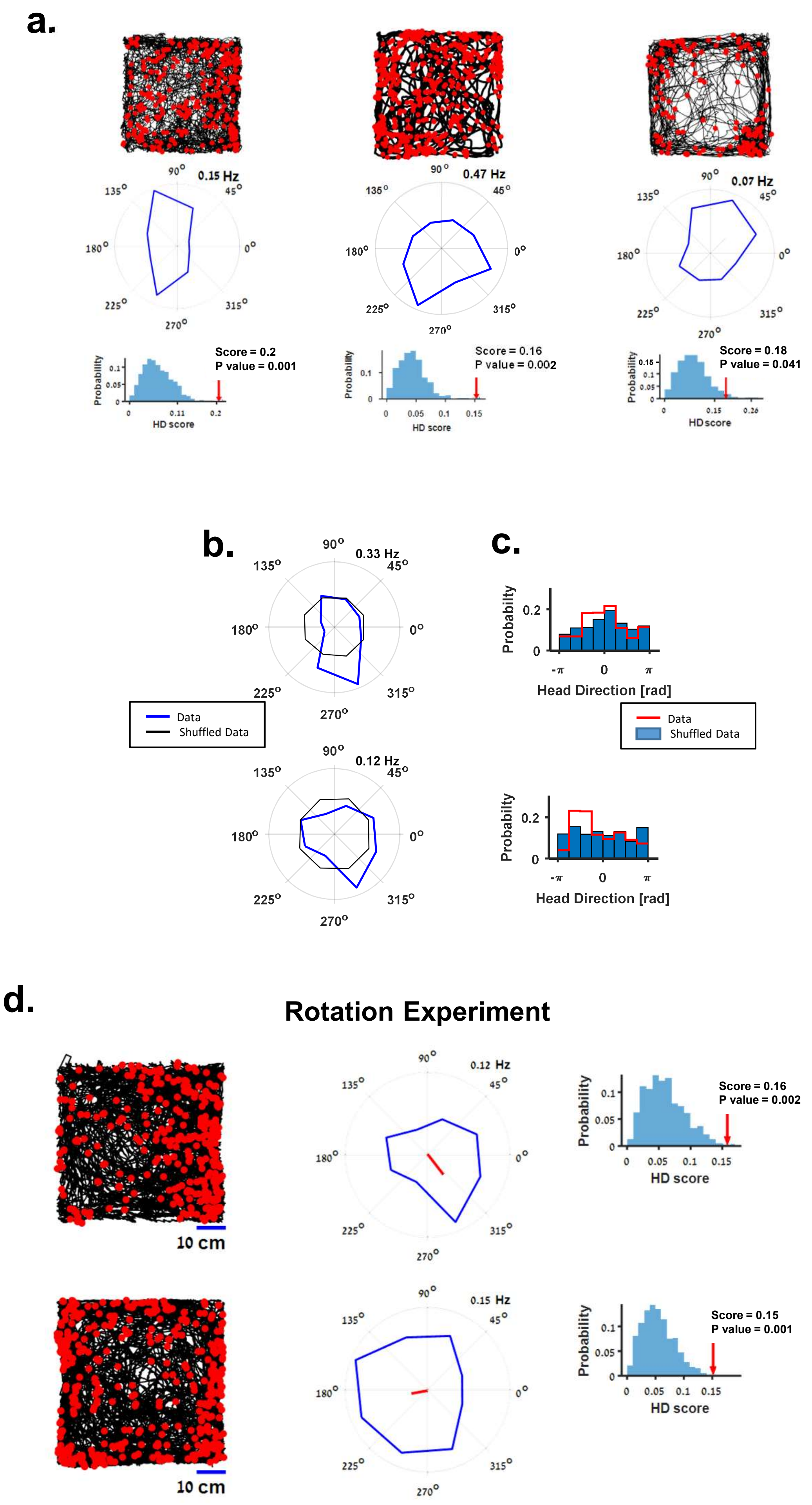

**Supplementary Figure 3| Head Direction Encoding Cells. a.** Additional examples of head direction cells. Each column of panels corresponds to a single cell. The cells were recorded in different sessions in different fish. For each cell, the trajectory (black curve, top panels) and action potentials (red dots) are presented in addition to the head direction tuning of the neuron (middle row panels). The statistical analysis shows that the head direction score of all cells was significant compared to chance (bottom panels). **b.** Average firing rate as a function of head direction (blue curve) for the two cells in Figure 3 a-h superimposed over shuffled data of the two cells. **c.** Probability of head direction during a spike time (red curve) for the two cells in Figure 3 a-h superimposed over shuffled data of the two cells. **d.** Visual cue rotation; we implemented two recording sessions, where before the second session, the local visual landmarks marked on the walls were rotated by 180 degrees. Cell activity showed 130$^o$ change in the directional preference corresponding to the rotation.

# Supplementary Figure S4 – Vinepinsky et al.
## **Speed encoding**

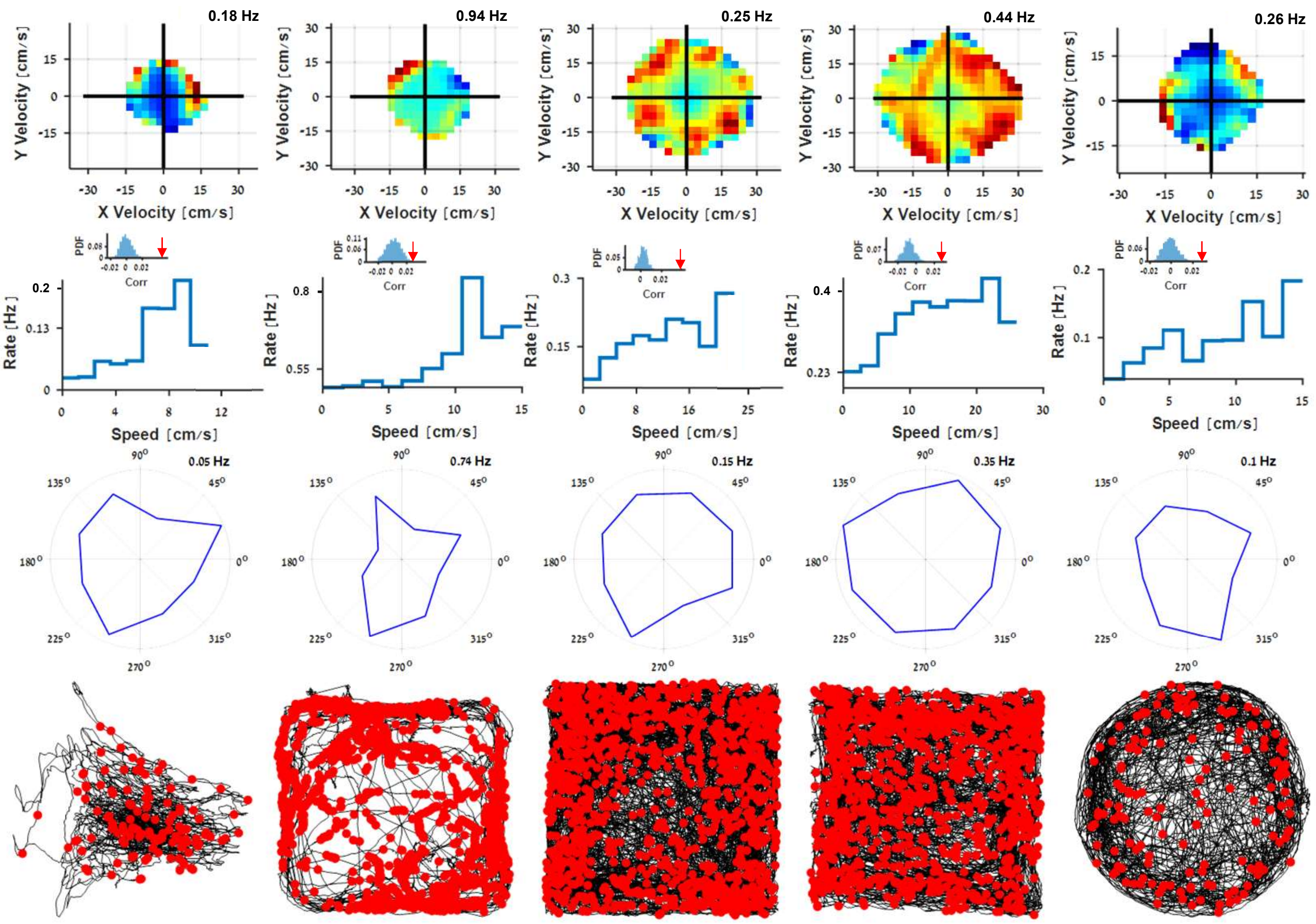

**Supplementary Figure 4| Speed Correlated Cells.** Additional examples of speed cells. The cells were recorded in different sessions in different fish. Each column of panels corresponds to a single cell. Top row, firing rate dependency on the velocity in two dimensions. Also presented is the rate as a function of speed (second row) together with the statistical analysis (second row, insets), firing rate tuning as a function of direction (third row) and the fish's trajectories (black curve, bottom row) and action potentials (red dots).

Supplementary Figure S5 – Vinepinsky et al.

**Velocity vector encoding**

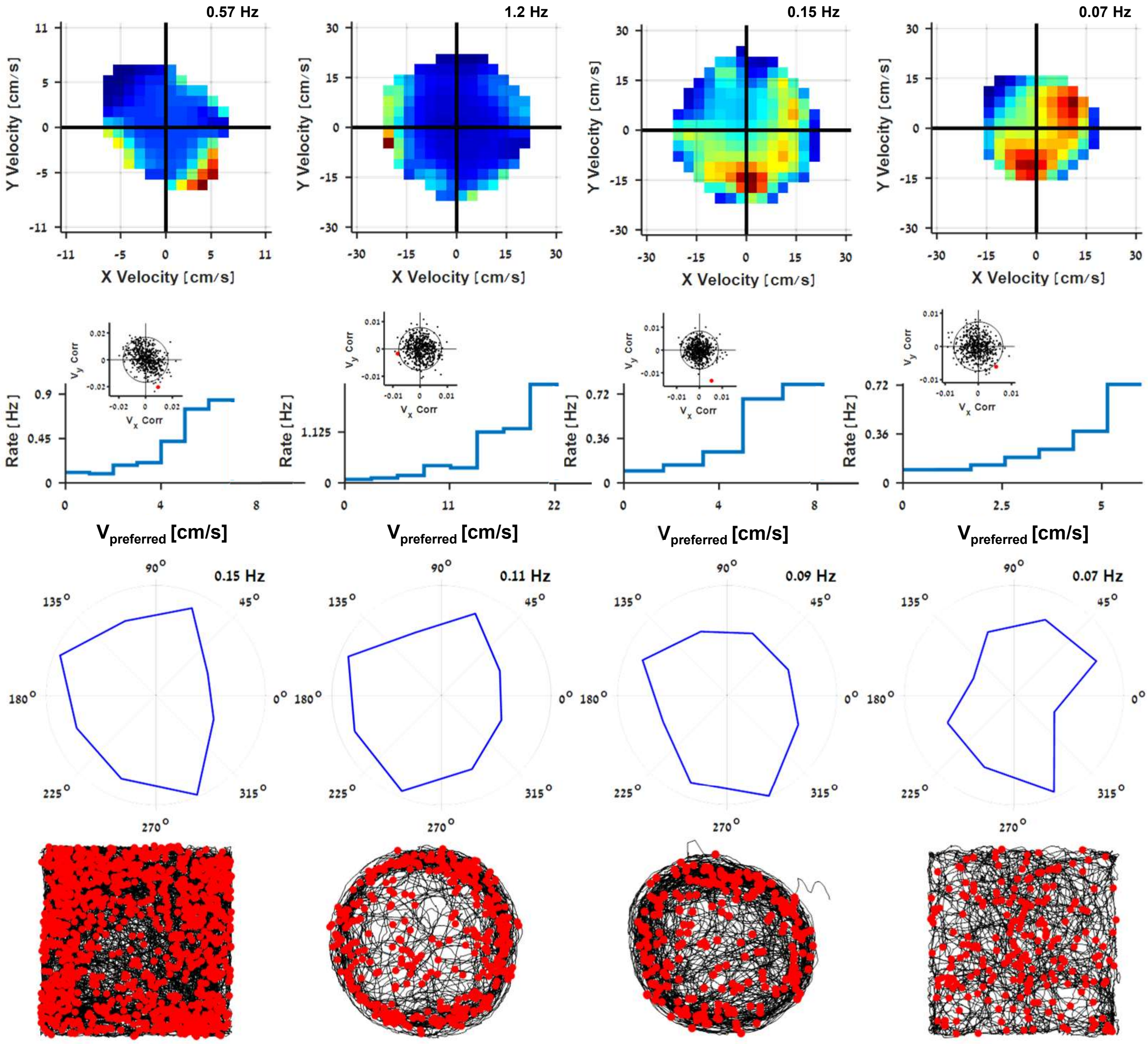

**Supplementary Figure 5| Velocity-Vector Encoding Cells.** Additional Examples of Conjunction Speed Cells and Head Direction; i.e. velocity cells. Each column of panels corresponds to a single cell. The velocity tuning of the cell is summarized in the color-coded firing rate map as a function of the two-dimensional velocity (top row). The dependence of the firing rate on speed along the one-dimensional projections of the preferred direction (second row) is shown together with a statistical analysis of the velocity tuned cells (second row, insets): the correlation coefficient between the spike train and the two Cartesian velocity components was calculated for shuffled spike trains (black dots) and the data (red dots). The 95% confidence circle is depicted. Third row: directional tuning of each cell. Bottom row: The fish's trajectories (black curve) and action potentials (red dots) recorded in each velocity cell. Waveform of the left column cell spiking activity is presented in Supplementary Figure 1g.

# Supplementary Figure S6 – Vinepinsky et al.
# **Velocity vector encoding controls**

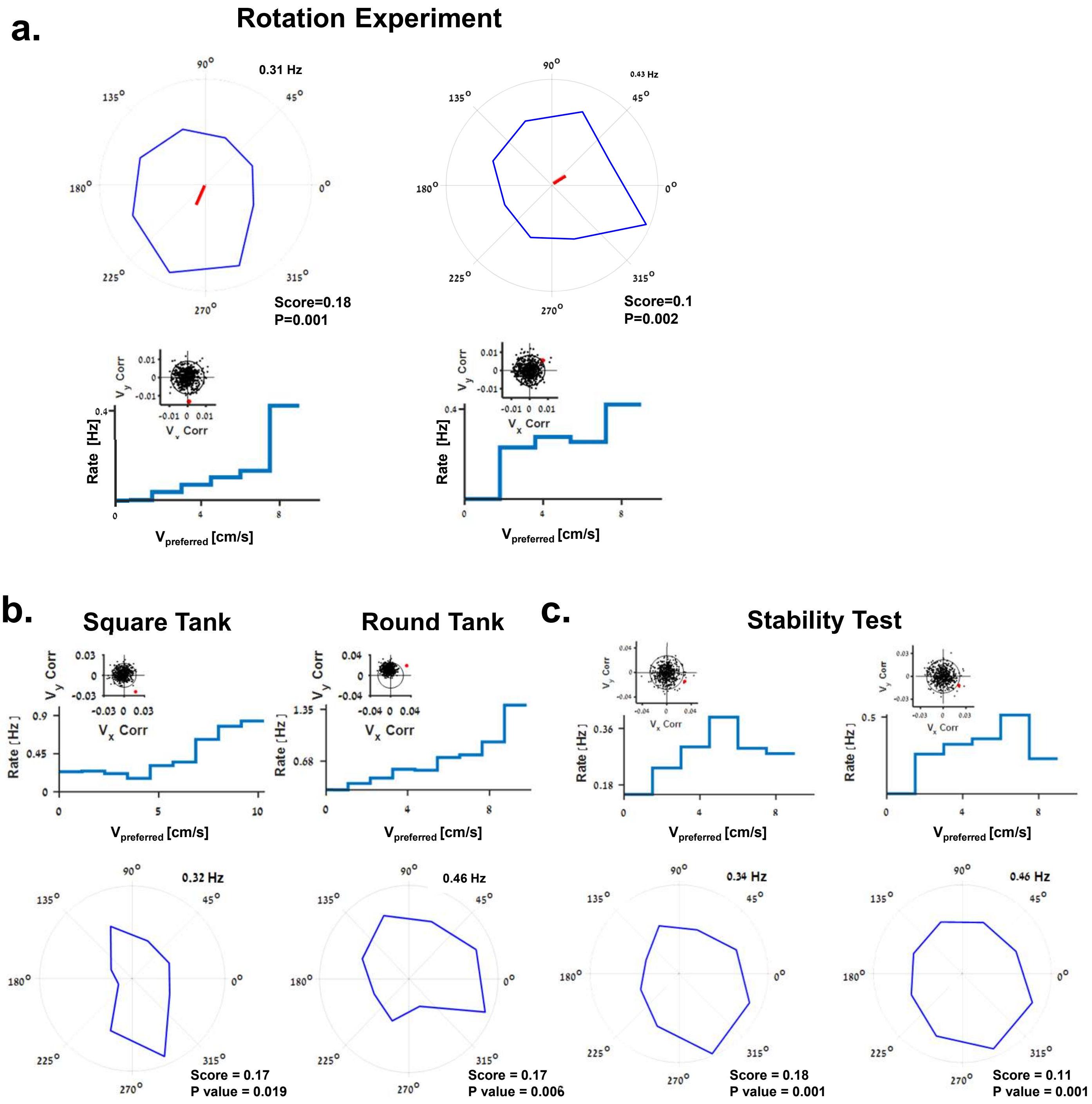

**Supplementary Figure 6| Velocity-Vector Cells- Control Experiments. a.** Visual cue rotation; we implemented two recording sessions where before the second session, the entire scene, with visual landmarks marked on the walls were rotated by 180 degrees. Cell activity showed a 150$^o$ change in directional preference corresponding to the rotation. **b.** Environment transfer. After recording in a square tank (left panels), the fish was transferred to a circular water tank (right panels). Cell activity shows both head direction preference and a correlation to velocity in both cases. Waveform of the cell spiking activity is presented in Figure 1d (blue cluster). Top row: firing rate tuning curve to velocities of the cell. Insets show the statistical analysis of the velocity tuned cells. Bottom row: Head direction tuning of the neuron. Head direction score and p values compared to chance are indicated on the bottom right side of panels. **c.** Split session analysis. Velocity cell activity in the first half (left panels) and the second half (right panels) of the same session. Cell shows both head direction preference and a correlation to velocity in both parts. Top row: firing rate tuning curve to velocity in each half of the experiment. Insets show statistical analysis of the velocity tuned cell. Bottom row: head direction tuning of the neuron.

Supplementary Figure S7 – Vinepinsky et al.

**Additional examples of recording locations**

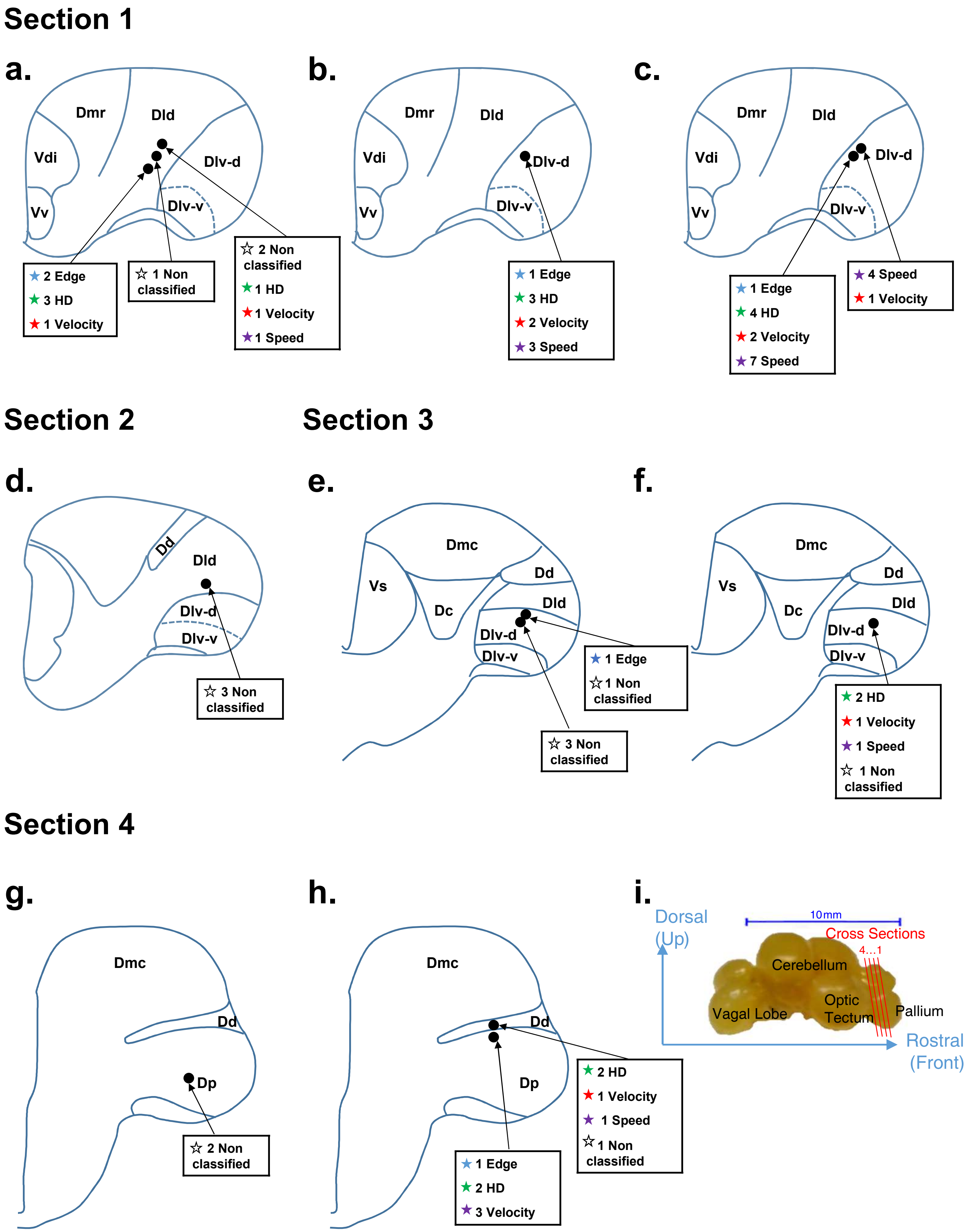

**Supplementary Figure 7| Additional Examples of Recording Locations. a-h.** Recording locations together with the classification of cells within these locations from 8 different fish. **i.** Goldfish brain structure. Cross sections 1-4 correspond to the section numbers indicated above for panels a, d, e and g.

Supplementary Figure S8 – Vinepinsky et al.

# Visual landmarks

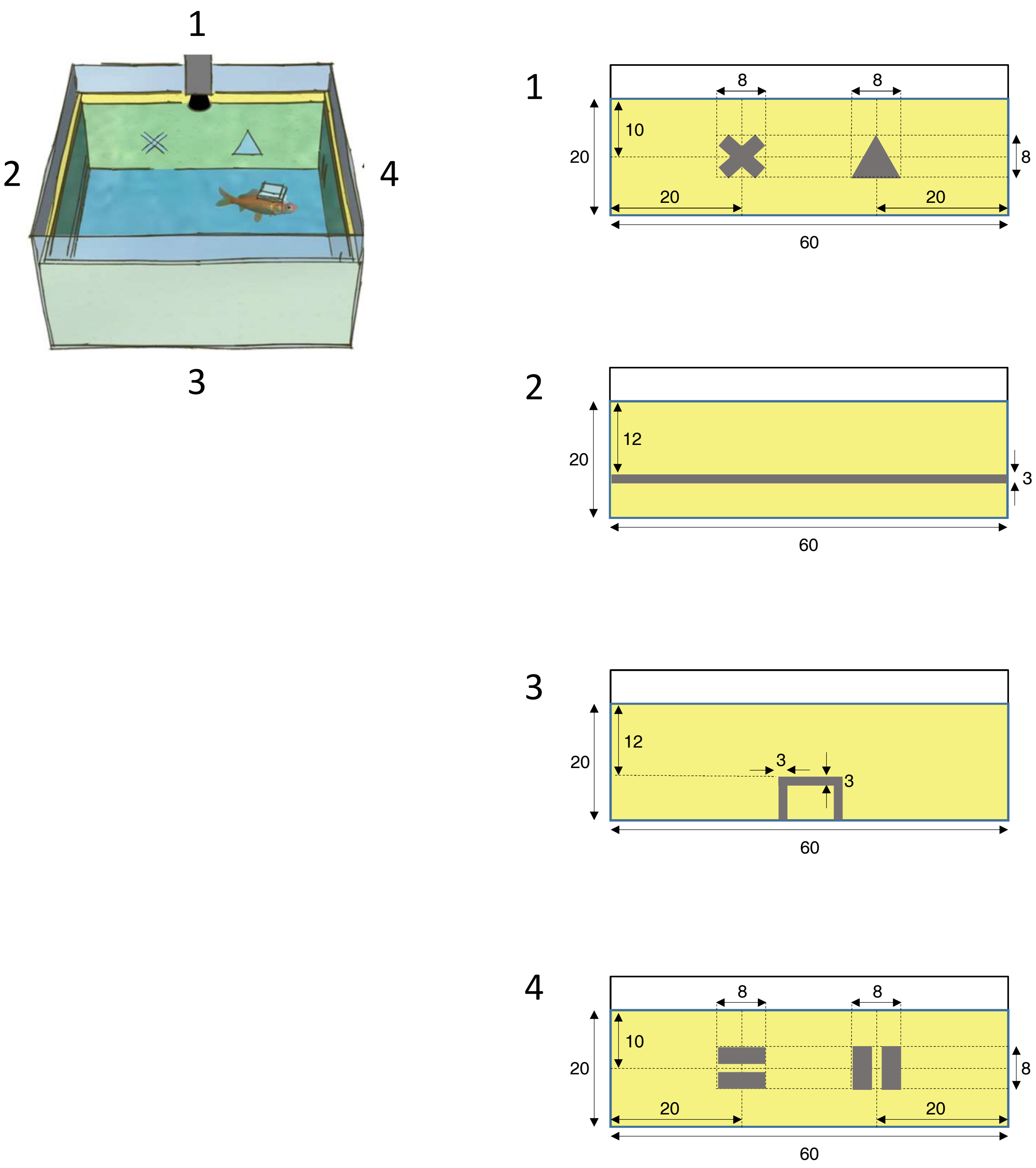


Units in [cm]

**Supplementary Figure 8| Visual Landmarks.** Schematic overview of the experimental water tank and visual cues painted on its walls.

Supplementary Figure S9 – Vinepinsky et al.

**Weakly spatially modulated cells hypothesis**

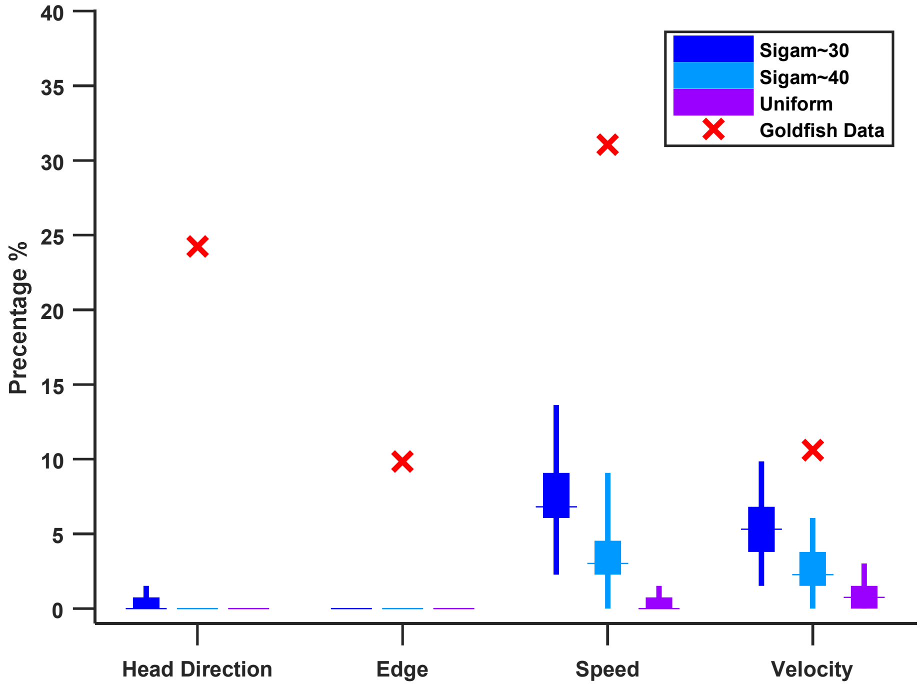

**Supplementary Figure 9| Testing for Weakly Selective Spatial Tuning Hypothesis**. In order to test whether a population of weakly selective spatial cells show the same edge, head direction and speed encoding properties as our data set we have simulated three types of data sets. The simulated data set cannot reproduce our results for different cell encoding properties (red X). To conclude, this analysis shows that our result cannot be explained by weakly spatially selective cell population alone.
